# A Gain-Of-Function Mutation in the *Plcg2* Gene Protects Mice from *Helicobacter felis*-Induced Gastric MALT Lymphoma

**DOI:** 10.1371/journal.pone.0150411

**Published:** 2016-03-11

**Authors:** Jennifer Gossmann, Manfred Stolte, Michael Lohoff, Philipp Yu, Roland Moll, Florian Finkernagel, Holger Garn, Cornelia Brendel, Alwina Bittner, Andreas Neubauer, Minh Q. Huynh

**Affiliations:** 1 Department of Hematology, Oncology and Immunology, Philipps University of Marburg, and University Hospital Giessen and Marburg, Marburg, Germany; 2 Institute of Pathology, Kulmbach Hospital, Kulmbach, Germany; 3 Institute of Medical Microbiology, Philipps University of Marburg, Marburg, Germany; 4 Institute of Immunology, Philipps University of Marburg, Marburg, Germany; 5 Institute of Pathology, Philipps University of Marburg, Marburg, Germany; 6 Institute of Molecular Biology and Tumor Research, Philipps University of Marburg, Marburg, Germany; 7 Institute of Laboratory Medicine and Pathobiochemistry—Molecular Diagnostics, Philipps University of Marburg, Marburg, Germany; University of Pittsburgh, UNITED STATES

## Abstract

Gastric mucosa-associated lymphoid tissue (MALT) lymphomas develop from a chronic *Helicobacter* infection. *Phospholipase C gamma 2* (*PLCG2*) is important for B-cell survival and proliferation. We used BALB/c mice with a gain-of-function mutation in the *Plcg2* gene *(Ali5)* to analyze its role in the development of gastric MALT lymphoma. Heterozygous BALB/c *Plcg2*^*Ali5/+*^ and wildtype (WT) mice were infected with *Helicobacter felis (H*. *felis)* and observed up to 16 months for development of gastric MALT lymphomas. In contrast to our initial hypothesis, *Plcg2*^*Ali5/+*^ mice developed MALT lymphomas less frequently than their WT littermates after long-term infection of 16 months. Infected *Plcg2*^*Ali5/+*^ mice showed downregulation of proinflammatory cytokines and decreased *H*. *felis*-specific IgG1 and IgG2a antibody responses. These results suggested a blunted immune response of *Plcg2*^*Ali5/+*^ mice towards *H*. *felis* infection. Intriguingly, *Plcg2*^*Ali5/+*^ mice harboured higher numbers of CD73 expressing regulatory T cells (Tregs), possibly responsible for impaired immune response towards *Helicobacter* infection. We suggest that *Plcg2*^*Ali5/+*^ mice may be protected from developing gastric MALT lymphomas as a result of elevated Treg numbers, reduced response to *H*. *felis* and decrease of proinflammatory cytokines.

## Introduction

Mucosa-associated lymphoid tissue (MALT) lymphomas are extranodal marginal zone B-cell lymphomas. There is a strong association between *Helicobacter pylori (H*. *pylori)* infection and MALT lymphoma, since *H*. *pylori* is detectable in 92–98% of gastric MALT lymphomas [1, 2] and cure of the infection leads to long-lasting remissions [3–7].

Several studies showed that different host gene polymorphisms in genes such as *IL-1*, *CTLA4* and *GST T1* are associated with increased inflammatory responses and consecutively with higher incidence of MALT lymphomas [8–11]. Previously, our group found that *phospholipase C gamma 2 (PLCG2)* is overexpressed in MALT lymphoma tissue [12]. In normal B-cells, B-cell receptor (BCR) antigen-binding results in PLCγ2 phosphorylation by Syk (spleen tyrosine kinase) and Btk (Bruton´s tyrosine kinase). Phosphorylated PLCγ2 is able to cleave phosphatidylinositol 4,5-bisphosphate (PIP_2_) into the second messengers inositol 1,4,5-trisphosphate (IP_3_) and diacylglycerol (DAG) [13]. IP_3_ is responsible for calcium release from the endoplasmatic reticulum, while DAG activates PKCβ (protein kinase C) and results in regulation of NF-κB and Ras signaling [13–16]. In turn, activation of NF-κB is responsible for cell differentiation, proliferation, survival and development of B-cells [15, 17].

The mouse strain *Plcg2*^*Ali5*^ *(Ali5*, *abnormal limb 5)* has a genomic gain-of-function mutation in the *Plcg2* gene, which results in Plcγ2 hyperactivity due to enhanced membrane adherence after BCR activation [18]. This point-mutation leads to symptoms of systemic inflammatory autoimmune diseases in *Plcg2*^*Ali5*^ mice, which show spontaneous swollen and inflamed paws and autoimmune lupus like disease symptoms, depending on the genetic background. In line with our mouse model, Ombrello et al. (2012) reported that patients with constitutive PLCγ2 activation show an autoimmune phenotype with cold urticaria, antibody deficiency and susceptibility to infection. Compared to the *Ali5* model, these patients harbour a deletion of the autoinhibitory region in the *Plcg2* gene [19].

In the present study, we examined the role of a gain-of-function mutation in the *Plcg2* gene in reference to the development of gastric B-cell lymphomas of the MALT-type. We hypothesized that due to their autoimmune-prone phenotype, mice with the mutated *Plcg2* gene were more susceptible to development of gastric MALT lymphomas. However, in contrast to our hypothesis, we observe less frequent transformation into MALT lymphomas in BALB/c *Plcg2*^*Ali5/+*^ mice as compared to wild-type (WT) littermates, and describe that this phenomenon correlates with impaired immune response, and elevated numbers of suppressive regulatory T-cells (Tregs).

## Materials and Methods

### Ethics statement

All animal experiments were performed in compliance with the German animal protection law. The study titled “*Helicobacter felis*-induced gastric MALT lymphoma in mice with a gain-of-function mutation in the *PLCg2* gene (MALT lymphoma development)”, were perfomed in approval with institutional guidelines and permissions by the local ethics committee (Regierungspräsidium Gießen) of the state of Hessen, Germany, under the permit numbers V54-19c 20-15(1) MR 20/11—Nr. 21/2009 and V54-19c 20 15h 01 MR 20/36 Nr. 77/2012. All efforts were made to minimize animal suffering and mice were killed by cervical dislocation.

### Animals

BALB/c wild-type (WT) mice were purchased from Harlan Winkelmann GmbH. *Plcg2*^*Ali5/+*^ mice with BALB/c background were kindly provided by the Institute of Immunology (Philipps-University Marburg, Germany). Mice were bred under standardized and specific pathogen-free conditions in air-conditioned rooms (temperature 22 ± 1°C, humidity 55 ± 5%) in IVC type II long cages filled with wood shavings under a 12 hours day-night cycle with lights on at 7:00 am. Animals had free access to autoclaved water and pellet food (Rod 18-R; LASvendi GmbH, Soest, Germany) was continuously available. We used heterozygous BALB/c *Plcg2*^*Ali5/+*^ mice for infection experiments, because homozygous *Plcg2*^*Ali5/Ali5*^ show strong inflammatory reactions on paws, eyes and internal organs and could not been used for long-term infection studies. During housing, infected animals were monitored 2–3 times a week for health status (e.g. piloerection, reduction of weight) and got points for it. Decision of euthanasia by cervical dislocation (after CO_2_ narcotization) was done, if animals got ≥ 7 points. The criteria list for decision of euthanasia is shown in S1 Table. Animals which died or have been killed due to the health status during the study (within 6 months after infection) were not included in the analysis. One mouse in group 1, 2 mice in group 3 and 5 mice in group 3 died unexpectedly within 6 months after infection and were not euthanized.

### Infection of mice

Three groups of female BALB/c *Plcg2*^*Ali5/+*^ and WT mice were orally infected with *H*. *felis*. Bacteria were harvested in sterile PBS and mice were gavaged with 2 x 10^8^ bacteria in 200 μl suspensions on days 1, 3 and 5. Mice were narcotized with CO_2_ before cervical dislocation and analyzed at 6, 12 and 16 months after infection (group 1: n = 11; group 2: n = 16; group 3: n = 17 per genotype). A fourth group was infected once with 2 x 10^8^ bacteria / mouse every 3rd month for 12 months (n = 11 per genotype). Uninfected animals served as controls. To confirm that mice were infected with comparable bacterial load, we performed quantitative PCR of stomachs from mice which were infected for 16 months. *H*. *felis* specific primers against 16S rDNA were used (see SI Material and Methods).

### Preparation of bacterial lysate

*Helicobacter felis* strain CS1 (ATCC49179) was purchased (American Type Culture Collection). *H*. *felis* was harvested in PBS, centrifuged at 3000 x g for 10 min and resuspended in 25 μl 1 x TES-Buffer (Sigma-Aldrich) plus 1 μl Ready-Lyse Lysozyme Solution (Epicentre Biotechnologies) and incubated for 60 min at room temperature. After a second centrifugation step at 4°C for 10 min and 20000 x g the supernatants were collected. Protein concentration was determined by BCA Protein Assay Kit (Thermo Fisher Scientific) according to the manufacturer´s instructions.

### Collection and preparation of mouse blood and tissue samples

RNA from whole blood was collected with RNAprotect Animal Blood Tubes and Kit (Qiagen) according to the manufacturer´s instructions. MNCs were prepared from spleen by disruption tissue with a pair of tweezers in sterile PBS, which were then processed for Ficoll-Hypaque density gradient centrifugation (FICOLL-Paque Plus, Amersham Biosciences). Residual red-blood cells were lysed by red-blood cell lysis buffer [155 mM NH_4_CL, 10 mM KHCO_3_, 0.1 mM EDTA, pH 7.5] for 5 min at room temperature. MLN cells were disrupted as described for spleen cells.

### Histology and histopathological examination

Stomach tissue was dissected along the small curvature, washed with NaCl and pinned flat on a cork board. After formalin fixation (4%), stomachs were embedded in paraffin. Series of 4 μm longitudinal sections were cut and stained with hematoxylin-eosin. Immunohistochemical stainings were conducted as previously described [12]. Following primary antibodies were used: monoclonal rat anti-mouse CD45R 1:200 (B220, clone RA3-6B2) (BD Pharmingen); polyclonal rabbit anti-cytokeratin 1:500 (catalogue number: Z0622; Dako GmbH). Immunohistochemical stainings were reviewed in a blinded manner by a reference pathologist (M.S.). Histology was examined as i) healthy gastric mucosa, ii) gastric mucosa with lymphoid aggregates indicating chronic gastritis, iii) gastric MALT lymphoma with infiltrates of centrocyte-like cells and replacement of gastric glands (including lymphoepithelial lesions (LEL) and lymphoepithelial destruction (LED)), according to Isaacson and Wright.[20]

Immunocytological CD3 and Foxp3 stainings were performed on formalin-fixed paraffin embedded (FFPE) spleens of uninfected and infected mice according to the avidin-biotin complex (ABC) method as previously described [21]. Following primary antibodies were used: anti-CD3 1:500 (clone SP7; Thermo Fisher Scientific) and anti-Foxp3 1:100 (clone FJK-16s; Affymetrix/eBioscience, Inc.). The total number of Foxp3^+^ Tregs, in 4–6 T-cell areas of the white pulp from each spleen, was determined microscopically in all cases by a single investigator to warrant homogeneity of data collection (Leica DMRB; Leica Microsystems GmbH). By using ImageJ v1.47 software (freeware, National Institutes of Health), the corresponding area of CD3^+^ T-cells was calculated and Foxp3^+^ Tregs were determined per 1000 μm^2^.

### RNA extraction and reverse transcription

Total RNA extraction from peripheral blood was prepared by using RNeasy Protect Animal Blood Kit (Qiagen). 1 μg of RNA was reverse transcribed to cDNA using Omniscript Reverse Transcription Kit (Qiagen). RT^2^ First Strand Kit (SABioscience) was used to transcribe 1 μg RNA from the blood of mice of group one for the performance of the RT^2^ Profiler PCR Array. All procedures were carried out according to the manufacturer´s guidelines.

### PCR Array of proinflammatory cytokines

To assess proinflammatory cytokines in mouse peripheral blood we used the RT^2^ Profiler PCR Array (SABioscience) according to the manufacturer´s instruction. The following amplification protocol was performed on the Mastercycler^®^ ep realplex 2 S (Eppendorf AG): 1 x 95°C for 10 min followed by 40 x (95°C for 15 s and 60°C for 1 min). Web-based software of SABioscience/Qiagen GmbH was used (http://pcrdataanalysis.sabiosciences.com/pcr/arrayanalysis.php).

### B-cell proliferation assay

Spleen B-cells were purified from MNCs by positive CD45R/B220 MACS MicroBead selection (Miltenyi Biotec) according to the manufacturer´s instruction. Purified B-cells were resuspended in BCM at 8 x 10^5^ cells/ml and were stimulated 48 h with 7.5 μg/ml LPS (Sigma-Aldrich), 5 μg/ml CpG-1826 5´-tccatgacgttcctgacgtt-3´ (TIB MOLBIOL Syntheselabor GmbH), 10 μg/ml α-IgM (Jackson ImmunoResearch Laboratories Inc.) and 10 μg/ml α-CD40 (clone HM40-3; BD Pharmingen) plus 10 ng/ml IL-4 (R&D Systems) or no stimulation. B-cells were pulsed with BrdU solution (final concentration of 10 μM) (BD Pharmingen) after 30 h. To determine proliferation, FITC BrdU Flow Kit and 7-AAD viability staining solution (BD Pharmingen) were used according to the manufacturer´s guidelines. Percentage of incorporated BrdU was measured by flow cytometry.

### Enzyme-linked immunosorbent assays

Serum concentration of total IgG1 and IgG2a were measured by ELISA as previously described [22]. Following primary antibodies were used: anti-mouse IgG1 (clone A85-3), anti-mouse IgG2a (clone R11-89) (BD Pharmingen). Serum samples were diluted 1:5000 (IgG1) and 1:2000 (IgG2a). Murine IgG1 (clone S1-68.1) and IgG2a (clone G155-178) (BD Pharmingen) were applied as standards in a 1:2 dilution series. Biotin-conjugated anti-mouse IgG1 (clone A85-1) and anti-mouse IgG2a (clone R19-15) (BD Pharmingen) were used as secondary antibodies.

For determination of *H*. *felis*-specific IgG1 and IgG2a antibody levels, ELISA plates were coated with soluble *H*. *felis* extract [10 μg/ml in PBS]. Serum samples were applied in a dilution of 1:100 (IgG1) and 1:10 (IgG2a). Secondary antibodies were used as previously described. Concentration of *H*. *felis*-specific IgG1 and IgG2a was estimated in relation to a pooled standard serum of infected mice in a titrated 1:4 dilution which was arbitrarily set at 10 labor units/ml [LU/ml].

### Flow cytometry

For IgG1 antibody production *in vitro*, B-cells were purified from mononuclear spleen cells by negative selection using CD43/Ly-48 magnetic-activated cell sorting MicroBeads as described by the manufacturer (Miltenyi Biotec). 3 x 10^5^ purified spleen B-cells/ml and 1 x 10^6^ total mLN cells/ml were resuspended in B-cell medium (BCM) containing 10% FCS, 1% Penicillin/Streptomycin, 1% Glutamin and 0.1% β-mercaptoethanol. Cells were stimulated for 4 days with 20 μg/ml LPS (Sigma-Aldrich), with and without 500 U IL-4 (R&D Systems). APC-conjugated antibody to IgG1 (clone M1-14D12) and PE-conjugated CD45R/B220 (clone RA3-6B2) antibody were purchased from Affymetrix/eBioscience, Inc. Total MNCs from spleen were used to analyze the number of regulatory T-cells and CD73 positive Tregs in *Plcg2*^*Ali5/+*^ and WT mice. Using the mouse regulatory T-cell staining Kit #2 (Affymetrix/eBioscience), cells were treated and stained according to manufacturer’s protocol. For CD73 staining, we purchased anti-CD73 (eFluor 450; clone TY/11.8) from Affymetrix/eBioscience. Flow cytometry was performed on FACS LSRII from BD Bioscience and results were analyzed with FlowJo software version 7.6 for Windows (Tree Star, Inc.).

### Statistical analysis

Differences in MALT lymphoma incidence was calculated with Fisher´s Exact Test. PCR array data were evaluated by the web-based analysis tool of SABioscience/Qiagen GmbH and calculated with t-test or two-way analysis of variance (ANOVA) (http://www.R-project.org). Statistical analysis of two-tailed t-test or Mann-Whitney U-test was performed with GraphPad Prism software version 5.01 (GraphPad Software, Inc.). Statistical significance was assigned at p ≤ 0.05.

## Results

### *H*. *felis*-infected *Plcg2*^*Ali5/+*^ mice are protected from the development of MALT lymphomas

**Fig 1:**
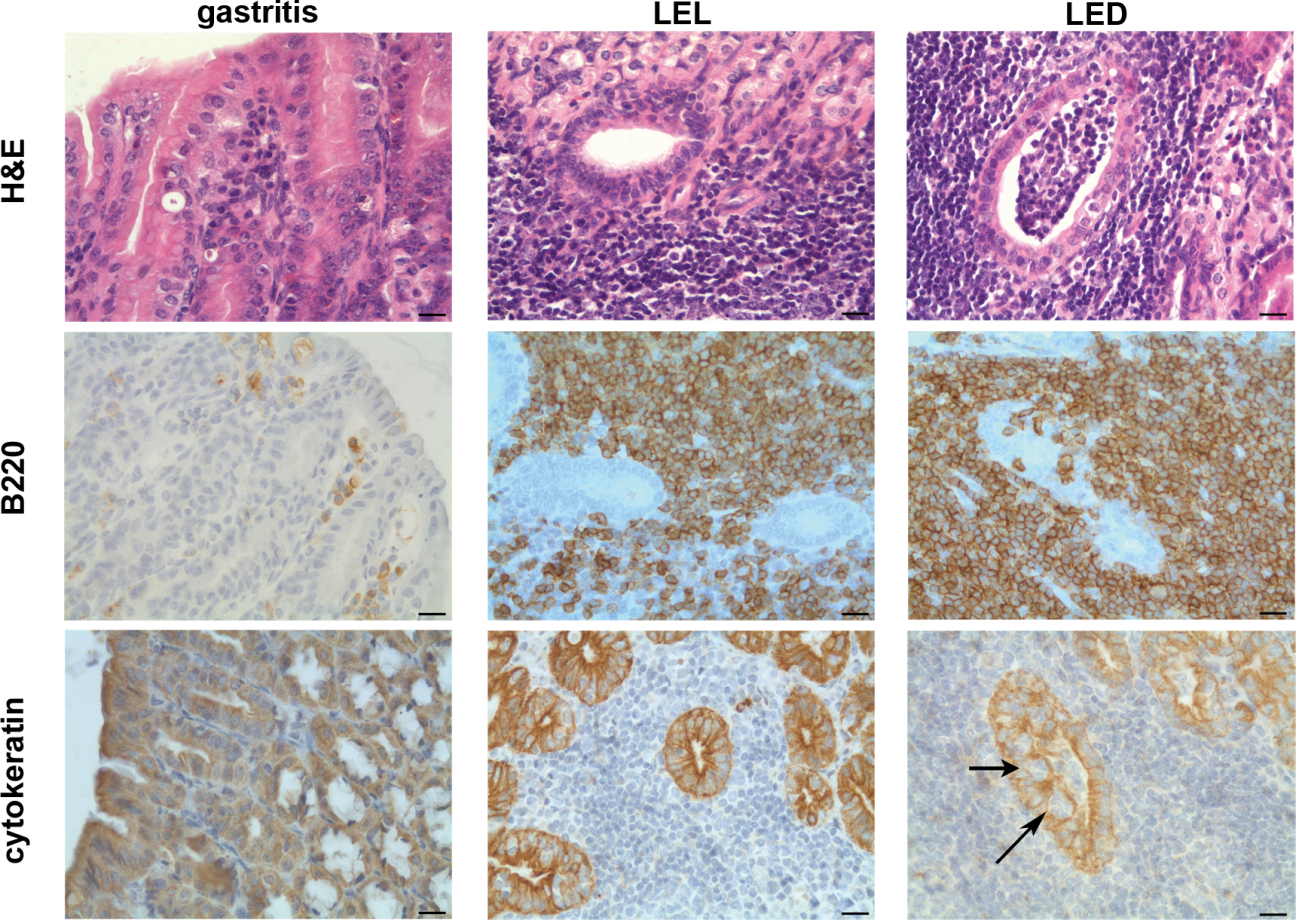
Histology and immunohistochemistry of gastric pathology. Histological and immunohistochemical staining of representative cases of chronic gastritis with lymphoid aggregates and MALT lymphoma with lymphoepithelial lesions (LEL) or lymphoepithelial destruction (LED); H&E staining, B220 labeling B-cells and anti-cytokeratine staining labeling the epithelium. Centrocyte-like cells infiltrate the gastric epithelium (arrows). Scale bars show 20 μm. Original magnification x40.

Host gene polymorphism influences the incidence of gastric MALT lymphomas [8–11]. We investigated whether a gain-of-function mutation in the *Plcg2* gene may have an effect on MALT lymphoma development after infection with *H*. *felis*. Three groups of *Plcg2*^*Ali5/+*^ and WT mice were infected and followed for 6, 12 and 16 months, respectively. In group one, no difference could be shown for both genotypes after 6 months of infection (Table 1, group 1): 6 out of 10 (60%) *Plcg2*^*Ali5/+*^ mice and 6 out of 11 (54%) WT mice (Fisher´s exact test, p = 1) developed chronic gastritis with lymphoid aggregates, but no gastric MALT lymphomas in either group. 12 months after infection (group 2), gastric MALT lymphomas were observed in 5 out of 15 (33%) mutant *Plcg2*^*Ali5/+*^ mice, as compared to 10 out of 15 (67%) (Fisher´s exact test p = 0.1431) in WT mice. At 16 months of follow-up (group 3), *H*. *felis-*infected *Plcg2*^*Ali5/+*^ mice showed significantly less gastric MALT lymphomas compared to their infected WT littermates (7 out of 14 (50%) vs. 14 out of 15 (93%)) (Fisher´s exact test, p = 0.0142). Non-infected mice developed neither gastritis nor MALT lymphomas (S2 Table). Fig 1 shows the histology of a representative mouse with chronic gastritis, LEL (lymphoepithelial lesions) as well as LED (lymphoepithelial destruction) as characteristics of MALT lymphomas. Subsequently, we investigated the bacterial load in both *H*. *felis* infected mice strains of group 3 by quantitative Real-Time PCR and could show that *Plcg2*^*Ali5/+*^ mice had the same bacterial load as compared to WT mice (S1 Fig). We conclude that the lower MALT lymphoma development in *Plcg2*^*Ali5/+*^ mice was not the cause of a lower colonization rate.

**Table 1:** Histopathological results of *H*. *felis*-infected Balb/c *Plcg2*^*Ali5/+*^ and WT mice.

Group	Time post-infection [months]	Genotype	Number of mice		Normal findings	Lymphoid aggregates	MALT lymphoma
			Enrolled^d^	Died^e^			
1^a^	6	*Plcg2* ^*Ali5/+*^	11	1	40% (4/10)	60% (6/10)	0%
		WT	11	0	46% (5/11)	54% (6/11)	0%
2^b^	12	*Plcg2* ^*Ali5/+*^	16	1	0%	67% (10/15)	33% (5/15)
		WT	16	1	0%	33% (5/15)	67% (10/15)
3^c^	16	*Plcg2* ^*Ali5/+*^	17	3	0%	50% (7/14)	50% (7/14)
		WT	17	2	0%	7% (1/15)	93% (14/15)

BALB/c *Plcg2*^*Ali5/+*^ and WT mice were orally infected with *H*. *felis* on day 1, 3 and 5 and followed-up for 6, 12 and 16 months. Normal finding indicates healthy gastric mucosa.

Statistical analysis was done with Fisher´s exact test for

^a,^ (comparison of genotypes for MALT lymphoma development with lymphoid aggregates) *Plcg2*^*Ali5/+*^ versus WT mice p = 1

^b,^ (comparison of genotypes for MALT lymphoma development with lymphoid aggregates) *Plcg2*^*Ali5/+*^ versus WT mice p = 0.1431

^c^ (comparison of genotypes for MALT lymphoma development with lymphoid aggregates) *Plcg2*^*Ali5/+*^ versus WT mice p = 0.0142

^d^ Number of mice enrolled at the beginning of the study.

^e^ Number of mice that died/killed during the study (within 6 months after infection).

We also asked whether repeated *H*. *felis* infection resulted in a higher prevalence of MALT lymphomas. To investigate this question, we re-infected a fourth group of mice in a 3 months interval for up to one year (Table 2). At the end of the experiment, we found that repeated infection with *H*. *felis* resulted in increased incidence of MALT lymphomas in both genotypes (83% *Plcg2*^*Ali5/+*^ mice vs. 100% WT mice) (Fisher´s exact test p = 1).

**Table 2:** Histopathological results of *H*. *felis* re-infected BALB/c *Plcg2*^*Ali5/+*^ and WT mice.

Group	Re-infection	Genotype	Number of mice		Normal findings	Lymphoid aggregates	MALT lymphoma
			Enrolled^a^	Died^b^			
4	4 times within 12 months	*Plcg2* ^*Ali5/+*^	11	1	40% (4/10)	60% (6/10)	0%
		WT	11	0	46% (5/11)	54% (6/11)	0%

BALB/c *Plcg2*^*Ali5/+*^ and WT mice were orally infected with *H*. *felis*. Mice were re-infected with *H*. *felis* in a 3 months interval for totally 12 months. Normal finding indicates healthy gastric mucosa.

Statistical analysis was done with Fisher´s exact test (comparison of genotypes for MALT lymphoma development with lymphoid aggregates) *Plcg2*^*Ali5/+*^ versus WT mice p = 1.

^a^ Number of mice enrolled at the beginning of the study.

^**b**^ Number of mice that died during infection time. A histological investigation of these mice was not possible.

### Proinflammatory genes are downregulated in *H*. *felis*-infected *Plcg2*^*Ali5/+*^ mice

To investigate whether the lower susceptibility of *Ali5* mice for MALT lymphoma development after *H*. *felis* infection is reflected in a differential gene expression profile, we performed an array testing for mRNA expression of 91 different proinflammatory genes. Peripheral whole blood samples of infected *Plcg2*^*Ali5/+*^ mice and WT littermates (followed-up for 6 months) as well as of uninfected mice were available for analysis. As expected for an autoimmune-prone mouse strain, non-infected *Plcg2*^*Ali5/+*^ mice revealed increases in the expression level of several proinflammatory cytokines and cell surface molecules as compared to uninfected WT mice (Table 3). Especially TNFSF13 (Tumor necrosis factor ligand superfamily member 13) and S100A8 (S100 calcium binding protein A8, calgranulin A) are described to be overexpressed in autoimmune diseases such as systemic lupus erythematodes (SLE) [18, 23]. However, this picture changed after *Helicobacter* infection. Surprisingly, 6 months after infection, several proinflammatory genes were downregulated in peripheral blood cells of *H*. *felis*-infected *Plcg2*^*Ali5/+*^ mice as compared to infected WT mice, including two important inflammatory cytokines, IFNγ and IL-1a. Within the *Ali5* genotype we also detected several genes which were downregulated in infected *Plcg2*^*Ali5/+*^ mice as compared to their uninfected *Plcg2*^*Ali5/+*^ littermates, such as the CXC chemokine subfamily members CXCL1, CXCL9 and CXCL10.

**Table 3:** Regulation of proinflammatory genes in whole peripheral blood of infected and uninfected mice.

Comparison	Genes	Mean Fold Regulation^*^	Mean p-value
Uninfected *Plcg2*^*Ali5/+*^ vs. uninfected WT	Cebpb	+2.187	0.0100
	Cxcl1	+2.851	0.0010
	Cxcr1	+3.997	0.0107
	Il-18	+5.613	0.0252
	S100a8	+2.614	0.0139
	Tlr2	+3.229	0.0024
	Tnfsf13	+3.742	0.0001
	Tnfrsf13c	-2.295	0.0140
	Tnfrsf17	-3.559	0.0209
Infected *Plcg2*^*Ali5/+*^ vs. infected WT	Ccl3	-2.841	0.0002
	Ccl4	-2.059	0.0037
	Ccl5	-2.331	0.0113
	Ccl20	-3.661	0.0292
	Cxcl1	-2.201	0.0153
	Ifng	-2.677	0.0421
	Il-1a	-2.898	0.0010
	Tnfrsf13c	-3.112	0.0052
Infected *Plcg2*^*Ali5/+*^ vs. uninfected *Plcg2*^*Ali5/+*^	Ccl3	-2.009	0.0498
	Ccl22	-2.372	0.0114
	Cxcl1	-2.187	0.0044
	Cxcl9	-2.175	0.0008
	Cxcl10	-2.069	0.0227
	Cxcr1	-2.326	0.0042
	Il-18	-5.676	0.0043
	Tlr2	-2.159	0.0005
	Tlr3	-2.059	0.0009
	Tnfsf13	-3.347	0.0003

Gene expression was determined by using RT^2^ Profiler PCR array data analysis v3.5 software. Statistical analysis was done with unpaired t-test (p ≤ 0.05). Uninfected mice: n = 4 per genotype; infected mice: n = 7 per genotype.

+, fold upregulation

-, fold downregulation (e.g. Cebpb is upregulated in uninfected *Plcg2*^*Ali5/+*^ mice and Tnfrsf13c is downregulated in uninfected *Plcg2*^*Ali5/+*^ mice).

^*^ Mean Fold Regulation of uninfected or infected mice.

The two-way ANOVA (analysis of variance) method was used to distinguish between gene profiles, which were dependent on the genotype or the infection status (S3 Table). According to the genotype status and independent of *H*. *felis* infection (*Plcg2*^*Ali5/+*^ versus WT mice), CCL3 (chemokine (C-C Motif) Ligand 3), CD40 and TNFRSF13c were downregulated, whereas CXCR1 (chemokine (C-X-C motif) receptor 1), S100A8 and TLR2 (toll-like receptor 2) were upregulated. Independent of the genotype, infection with *H*. *felis*, resulted in downregulation of several genes, such as CXCL5 and TNFRSF13c (infected versus uninfected mice).

To validate the array results, we performed quantitative PCR analysis for TNFRSF13c and S100A8, which were the most significant regulated genes. By this, we confirmed the data shown in S3 Table that these genes were significantly regulated, dependent on genotype status (S4 Table).

### Protection from MALT lymphoma in *Plcg2*^*Ali5/+*^ mice is not a result of a distinct B-cell defect

**Fig 2:**
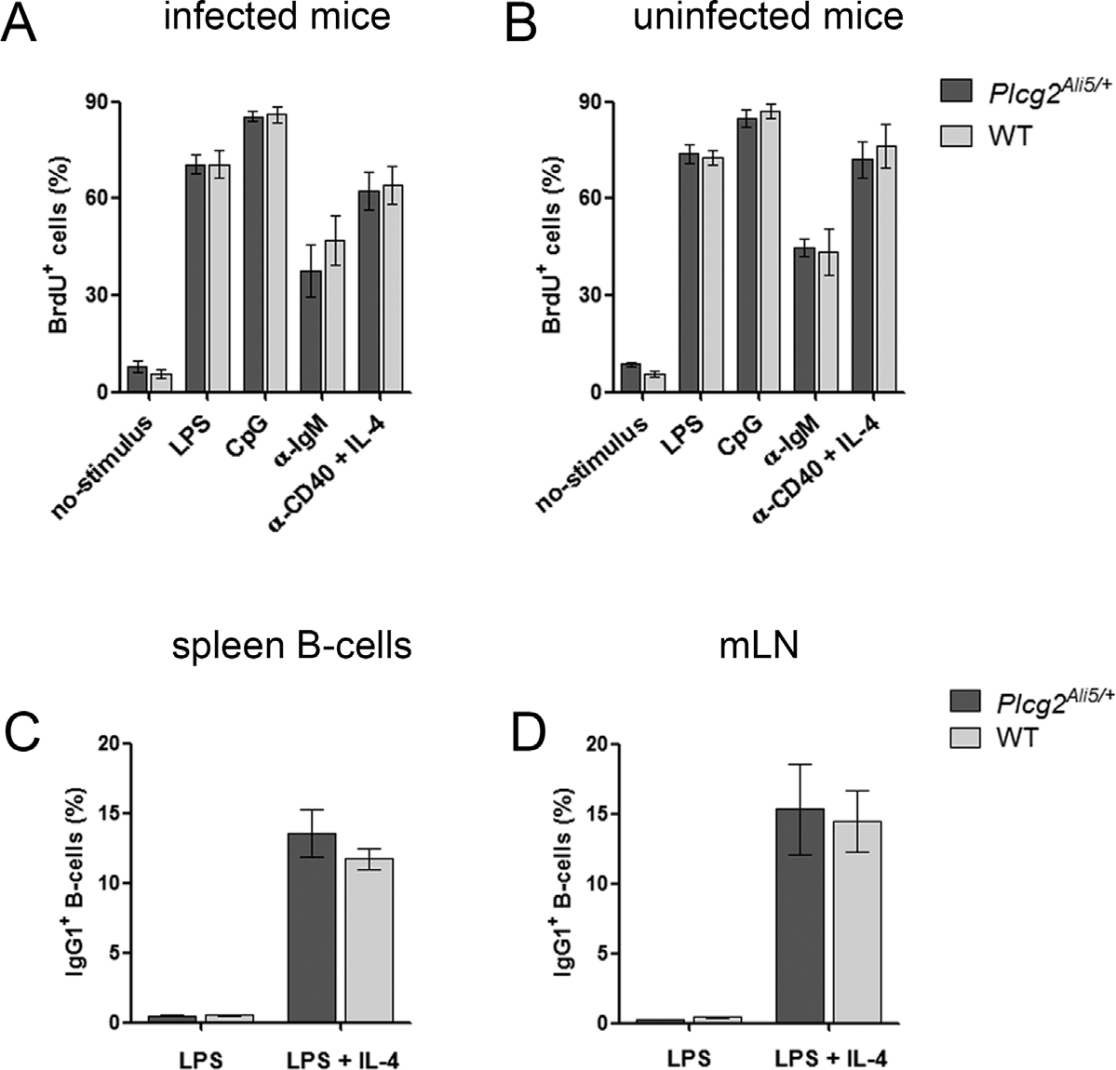
*Plcg2*^*Ali5/+*^ mice show no B-cell defect. (A-B) Proliferation of primary spleen B-cells was measured using the BrdU flow Kit. Purified CD45R/B220^+^ B-cells were stimulated for 48 h with LPS, CpG, α-IgM or α-CD40 + IL-4 or left unstimulated. Cell proliferation was assessed by flow cytometry after pulse with BrdU for the last 18 h of culture. Stimulation of primary spleen B-cells of (A) *H*. *felis*-infected mice (infection time for 12 weeks) or (B) uninfected mice showed no differences in cell proliferation. Data show live cells as mean ± SEM including n = 6 infected mice/genotype and n = 3 uninfected mice/genotype. (C-D) IgG1 class switching through stimulation of B-cells of non-infected mice *in vitro*. Purified CD43^-^ spleen B-cells and total mLN cells were stimulated with LPS, with or without IL-4. 4 days after stimulation cells were stained with anti-IgG1 and B-cells were counterstained with anti-CD45R/B220. Cells were analyzed by flow cytometry and membrane IgG1 positive B-cells of (C) spleen and (D) mLN B-cells are shown as percentages. B-cells treated with LPS only were used as controls. Data represent mean ± SEM of 2 independent experiments including n = 6 individually examined mice/genotype. All statistical analyses were done by unpaired Student´s t-test. mLN = mesenteric lymph nodes.

To investigate, whether defective *Plcg2*^*Ali5/+*^ B-cells were responsible for impaired MALT lymphoma development and downregulation of proinflammatory genes, we performed several experiments to analyze different aspects of B-cell function. To test for proliferation, purified spleen CD45R/B220^+^ B-cells from *H*. *felis*-infected and uninfected mice were used for *in vitro* stimulation with different mitogens. S-phase B-cells (BrdU^+^ cells) were used to analyze proliferating cells. We could not detect any differences between the percentages of S-phase B-cells with different stimulation agents in infected and uninfected cells (Fig 2A and 2B). Additionally, we observed comparable B-cell proliferation in *Plcg2*^*Ali5/+*^ and WT mice after BCR stimulation.

Next, we investigated *in vitro* whether B-cells of uninfected *Plcg2*^*Ali5/+*^ mice show a physiological reaction to LPS and IL-4. It is known that activation of B-cells with LPS and IL-4 stimulation results in class switching from IgM to IgG1 [24, 25]. Therefore we analyzed membrane IgG1 expression on B-cells after stimulation. Expression of membrane IgG1 on B-cells was inducible in both *Plcg2*^*Ali5/+*^ and littermate B-cells after LPS and IL-4 mediated class switching (Fig 2C and 2D).

### *Plcg2*^*Ali5/+*^ mice show an impaired *H*. *felis*-specific antibody response

**Fig 3:**
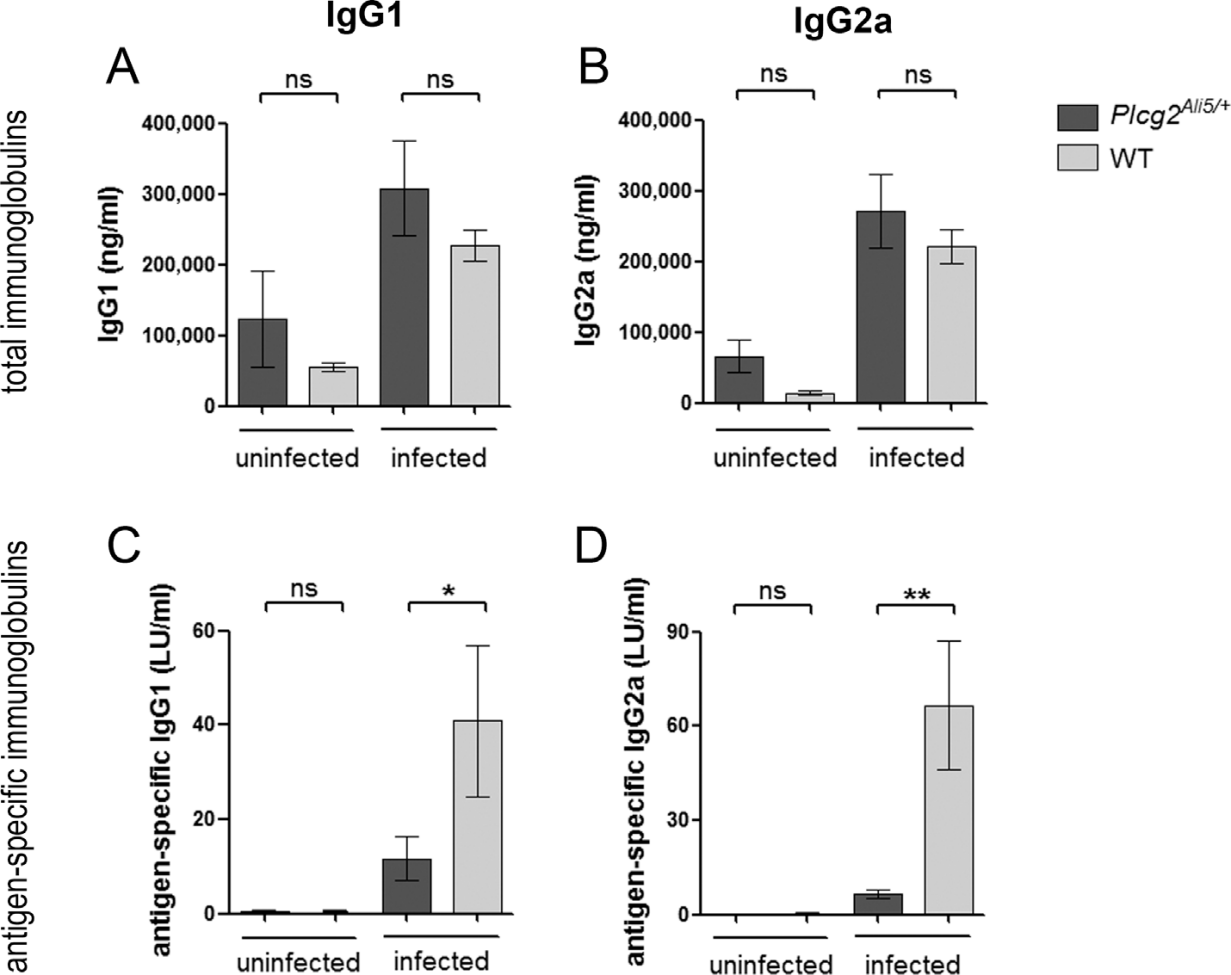
Total and *H*. *felis*-specific immunoglobulin levels in infected and uninfected mice. Sera from infected mice were collected 12 weeks after infection with *H*. *felis* and antibody titers were determined by ELISA. (A-B) Total immunoglobulin levels were elevated to similar extent in both infected genotypes as compared to their uninfected littermates while (C, D) *H*. *felis*-specific immunoglobulin levels were decreased in infected *Plcg2*^*Ali5/+*^ mice. Data represent mean ± SEM of 3 independent experiments including n = 14 to 15 infected *Plcg2*^*Ali5/+*^ mice and n = 15 infected WT mice. Two independent experiments were done for uninfected mice (n = 4 per genotype). Data were analyzed by using the Mann-Whitney U-test. LU = labor units; ns = not significant. *p ≤ 0.05; **p ≤ 0.01.

After having examined the inducibility of an Ig class switch in *Plcg2*^*Ali5/+*^ and WT B-cells *in vitro* by analyzing membrane bound immunoglobulins, we sought to determine *in vivo* titers of serum antibodies. 12 weeks after *H*. *felis* infection, sera from both genotypes were obtained and total and antigen-specific immunoglobulin levels were analyzed by ELISA (Fig 3). After infection, total serum IgG1 and IgG2a levels were elevated in both infected genotypes as compared to their uninfected littermates (Fig 3A and 3B). Concerning *H*. *felis*-specific IgG1 and IgG2a, no antibodies could be detected in uninfected mice of both genotypes, demonstrating that crossreactivity to any other *Helicobacter* species potentially present in the microflora played no role in our investigation. Specific antibodies appeared after *H*. *felis* infection in both genotypes, but their titers were significantly lower in infected *Plcg2*^*Ali5/+*^ animals. *H*. *felis*-infected *Plcg2*^*Ali5/+*^ mice showed 3.5-fold lower IgG1 (p = 0.0307) and 10-fold lower IgG2a levels (p = 0.0016) in comparison to their infected WT littermates (Fig 3C and 3D).

### Protection from MALT lymphoma correlates with higher frequency of regulatory T-cells

**Fig 4:**
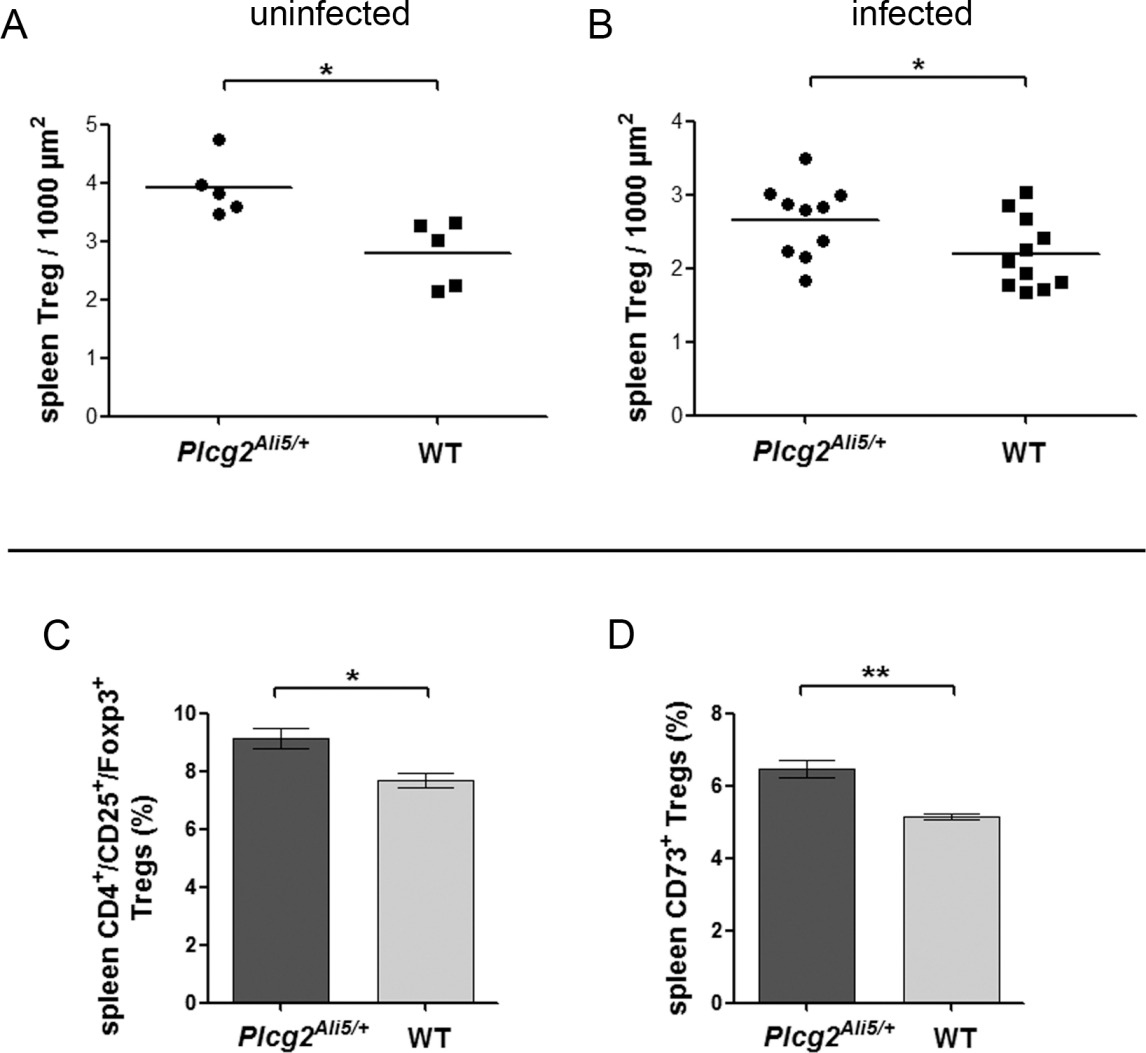
Increased Foxp3^+^ Treg numbers in spleen tissue of BALB/c *Plcg2*^*Ali5/+*^ mice. Histological scoring of Foxp3^+^ Tregs in spleen of (A) uninfected and (B) infected mice. *Plcg2*^*Ali5/+*^ and WT mice (n = 5 per genotype for uninfected mice; n = 10 infected *Plcg2*^*Ali5/+*^ mice, n = 11 infected WT mice) were used to count Foxp3^+^ Tregs in the T-cell area of the white pulp. Foxp3^+^ Tregs were determined on a CD3^+^ T-cell area of 1000 μm^2^. (C-D) Relative proportion of Tregs and ecto-5`-nucleotidase (CD73) expressing Tregs of CD4^+^ spleen cells from uninfected *Plcg2*^*Ali5/+*^ and WT mice. Mouse spleen MNCs were stained with *Mouse Regulatory T-cell Staining Kit #2* and co-stained with anti-CD73. Cells were analyzed by flow cytometry for CD4^+^/CD25^+^/Foxp3^+^ Tregs and CD73^+^ expressing Tregs determined from CD4^+^ T-cells. A representative of two to three independent experiments with three to four mice per genotype is shown. Distribution of (C) CD4^+^/CD25^+^/Foxp3^+^ Tregs and (D) CD73^+^ expressing Tregs in *Plcg2*^*Ali5/+*^ and WT mice are shown in percentages. Data represent mean ± SEM of 2 to 3 independent experiments and were calculated using Student´s t-test. MNCs = mononuclear cells; Tregs = regulatory T-cells. *p ≤ 0.05; **p ≤ 0.01.

Depletion of Tregs in *Helicobacter*-infected mice results in increased gastric inflammation [26]. Higher Treg numbers could explain an impaired immune response towards *H*. *felis*. Therefore we measured numbers of Treg in spleen tissue. By using immunohistochemical staining of CD3 and Foxp3 in T-cells of uninfected and infected *Plcg2*^*Ali5/+*^ and WT mice, we determined the number of Foxp3^+^ CD3^+^ Tregs per 1000 μm^2^ of area composed of CD3^+^ T-cells (S2 Fig). Our results revealed significantly more Tregs in spleen tissue of uninfected and infected *Plcg2*^*Ali5/+*^ mice as compared to WT mice (p = 0.0109 and p = 0,0451) (Fig 4A and 4B).

It was previously shown that CD73 expressing Tregs suppress *H*. *felis*-induced gastritis in mice [27]. Based on these data, we focused on CD73^+^ Tregs. We analyzed the percentages of CD73^+^ Tregs among the CD4^+^/CD25^+^/Foxp3^+^ Treg population in spleen mononuclear cells (MNCs) of uninfected *Plcg2*^*Ali5/+*^ and WT mice (Fig 4C and 4D). We could confirm our immunohistochemical results by flow cytometry and found that *Plcg2*^*Ali5/+*^ mice harboured a significantly higher percentage of Tregs (mean = 9.13% of CD4^+^ cells) as compared to WT mice (mean = 7.68% of CD4^+^ cells) (*p* = 0.0125) (Fig 4C). Furthermore, *Plcg2*^*Ali5/+*^ mice were expressing more CD73^+^ Tregs (mean = 6.48% of CD4^+^ cells) compared to their WT littermates (mean = 5.16% of CD4^+^ cells) (p = 0.0017) (Fig 4D).

## Discussion

We have found that *H*. *felis*-infected *Plcg2*^*Ali5/+*^ mice showed significantly less gastric MALT lymphomas compared to their WT littermates after 16 months of infection. These results were somehow unexpected, because we had assumed that *Plcg2*^*Ali5/+*^ mice might show a stronger gastric inflammatory response and thus an increased induction of MALT lymphomas after *H*. *felis* infection. This seemed likely, as Plcg2 activates NF-κB [16], a key molecule of MALT lymphoma development [28]. Furthermore, we found that repeated infection with *H*. *felis* resulted in increased incidence of MALT lymphomas in both genotypes and after 12 months of follow-up differences were no longer detectable. We suppose that mice infected regularly eliminate, at least in part, *H*. *felis* over a longer period of time and the inflammatory response can diminish. However, repeated infection with *H*. *felis* may result in a prolonged inflammatory stimulus that yields earlier and more MALT lymphoma development. This result emphasizes that development of gastric MALT lymphomas is strongly dependent on chronic *Helicobacter* infection and that repeated antigen-stimulation can overcome the influence of a protective host-related gene polymorphism.

We verified that uninfected BALB/c *Plcg2*^*Ali5/+*^ mice show an increased proinflammatory cytokine profile in the peripheral blood. These results were in accordance with previously published data for heterozygous *Plcg2*^*Ali5/+*^ mice with C3H background [18]. Especially TNFSF13 (Tumor necrosis factor ligand superfamily member 13) and S100A8 (S100 calcium binding protein A8, calgranulin A) are described to be overexpressed in autoimmune diseases such as systemic lupus erythematodes (SLE) [18, 23]. However, in our model, this picture changed after *Helicobacter felis* infection. In comparison to WT littermates, *H*. *felis*-infected *Plcg2*^*Ali5/+*^ mice showed less induction of cytokines, such as CCL3, CCL4, CCL5, CCL20 and CXCL1. We did not find a direct link between PLCG2 and cytokine production. Thus, these cytokines are known to be produced by gastric epithelial cells as a result of a Helicobacter infection [29–36]. In addition, *H*. *pylori* infection induces infiltration of IFNγ producing CD4^+^ TH1 cells in the gastric mucosa, which is responsible for the increased mucosal inflammation and destruction [37, 38]. Also the proinflammatory cytokine IL-1a, which is known as an acute phase cytokine, plays an important role in early inflammation and is mainly produced by macrophages [39]. *H*. *felis*-infected *Plcg2*^*Ali5/+*^ mice also showed less production of these two important inflammatory cytokines.

Less induction of cytokines in *H*. *felis*-infected *Plcg2*^*Ali5/+*^ mice may be a hint for an impaired recognition of these mutated mice toward *H*. *felis*. In fact, we found that *H*. *felis* antigen-specific IgG1 and IgG2a titers in sera of *Plcg2*^*Ali5/+*^ mice were significantly decreased.

The inadequate immune response to *H*. *felis* infection was not due to a general B-cell defect in BALB/c *Plcg2*^*Ali5/+*^ mice. Therefore, our findings argue against a simple B-cell defect in *Plcg2*^*Ali5/+*^ mice as being decisive for reduced MALT lymphoma development.

Rad et al. have previously shown that depletion of Tregs in *Helicobacter* infected C57BL/6 mice by an anti-CD25 mouse antibody results in severe gastritis and increased antigen-specific antibody titers [26]. Therefore we speculated that, *vice versa*, elevated Treg numbers in *Plcg2*^*Ali5/+*^ mice could lead to decreased inflammatory response to Helicobacter infection and, as we have shown, decreased *H*. *felis*-specific antibody titers. In keeping, we show that *Plcg2*^*Ali5/+*^ mice harbour significantly higher numbers of CD4^+^/CD25^+^/Foxp3^+^ Tregs in spleen tissue as compared to WT littermates. Kaparakis et al. have also shown increased *H*. *felis-*specific antibody titers after Treg depletion [40]. Tregs are able to inhibit B-cell antibody responses dependent or independent of T-cell stimulation [41]. Suppression of TH1/TH2 cells might be a possible effect that is responsible for reduced antigen-specific antibody titers in *Plcg2*^*Ali5/+*^ mice.

There are conflicting data about the incidence of Tregs in inflammatory-autoimmune diseases. Some studies showed lower numbers of Tregs in autoimmune individuals with SLE [42, 43] and other studies found even more Tregs in autoimmune patients with rheumatoid arthritis, SLE and Sjögren´s syndrome [44–49]. Yan et al. have shown that the immunosuppressive Treg function in SLE patients is impaired by IFNα-producing antigen-presenting cells (APCs). However, *in vitro* experiments show that Tregs of SLE patients are not per se dysfunctional. Tregs exhibited normal activity, if cultured with normal donor APCs [44]. These publications support our findings of both, elevated Treg numbers and increased proinflammatory cytokines in uninfected *Plcg2*^*Ali5/+*^ mice. We suggest for our mouse model that the *H*. *felis* infection reconstitute the immunosuppressive function of Tregs. However, the exact regulatory mechanism is undetermined.

One of the most important factors for suppression of T-cell-mediated immune responses is the enzyme ecto-5`-nucleotidase (CD73). CD73 is expressed particularly by Tregs, but also by tumor cells, myeloid-derived and endothelial cells. This enzyme is known to generate anti-inflammatory adenosine that protects tissue damage [50–54]. The importance of CD73 expression was demonstrated in a study by Alam and colleagues [27]. They showed that *H*. *felis* infected CD73-deficient mice have increased levels of proinflammatory cytokines and severe gastritis [27]. These data are in line with our findings, that *Plcg2*^*Ali5/+*^ mice had not only higher numbers of CD4^+^/CD25^+^/Foxp3^+^ Tregs in spleen tissue as compared to WT mice, but also higher numbers of CD73^+^ Tregs. Our results support the hypothesis of Kaparakis et al. that Tregs have a potential role in inhibition of lymphoma development after a lengthy infection time with *Helicobacter*.

Although *Helicobacter spp*. induce specific immune responses, the immune system of the host often fails to clear the infection and the bacterium can persist lifelong. Interaction of bacterial virulence factors and specific immune answer of the host influence the outcome of the infection. Several studies showed that different host gene polymorphisms in genes such as IL-1, CTLA4 and GST T1 are associated with increased inflammatory responses and consecutively with higher incidence of MALT lymphomas [8–11]. Our mouse model shows for the first time that a gene polymorphism could also be responsible for protection from MALT lymphoma development.

In conclusion, we found hints that elevated Treg numbers in *Plcg2*^*Ali5/+*^ mice play a decisive role in the outcome of *Helicobacter* infection. Although Plcγ2 is normally involved in activation of immune cells, we could show that *Plcg2*^*Ali5/+*^ mice were protected from developing gastric MALT lymphomas after long-term infection with *H*. *felis*, most likely as a result of increased numbers of immunosuppressive CD73^+^ Tregs.

## Supporting Information

S1 ARRIVE Checklist The ARRIVE Guidelines Checklist. (DOCX)

S1 Fig *Plcg2*^*Ali5/+*^ and WT mice of group 3 had the same *H*. *felis* colonization rate. The bacterial load in gastric tissue of *Plcg2*^*Ali5/+*^ (n = 10) and WT mice (n = 12) was measured by quantitative Real-Time PCR, 16 months after *H*. *felis* infection. Data represent mean ± SEM and were calculated using Student´s t-test. ns = not significant.
(TIF)

S2 Fig CD3 and Foxp3 immunohistochemistry of a murine spleen. A representative (A-B) CD3 and (C-D) Foxp3 immunohistochemical staining of a spleen of an uninfected mouse. The same five T-cell areas (in CD3 and Foxp3 staining) of the white pulp were determined microscopically and total Foxp3^+^ Tregs were determined with a magnification of x40. By using ImageJ v1.47 software total CD3 area was calculated (thin yellow line) and Foxp3^+^ Tregs were determined in relation to 1000 μm^2^ (CD3^+^ T-cell area). (A and C) Scale bars show 200 μm with an original magnification of x2,5 or (B and D) 20 μm with an magnification of x40.
(TIF)

S1 Materials and Methods (DOCX)

S1 Table Scoring System. (DOCX)

S2 Table Histopathological results of uninfected Balb/c *Plcg2*^*Ali5/+*^ and WT mice. (DOCX)

S3 Table Proinflammatory cytokine profile of *Plcg2*^*Ali5/+*^ and WT mice (either uninfected or 6 months after *H*. *felis* infection) grouped in genotype and infection status. (DOCX)

S4 Table PCR array validation by quantitative PCR. (DOCX)
