# Supplementary material for: A Gain-Of-Function Mutation in the Plcg2 Gene Protects Mice from Helicobacter felis-Induced Gastric MALT Lymphoma
Source: PLoS One. 2016 Mar 11;11(3):e0150411. doi: 10.1371/journal.pone.0150411 (PMC4788355; doi:10.1371/journal.pone.0150411)
Supplement: S1 ARRIVE Checklist — (DOCX) [file pone.0150411.s001.docx]

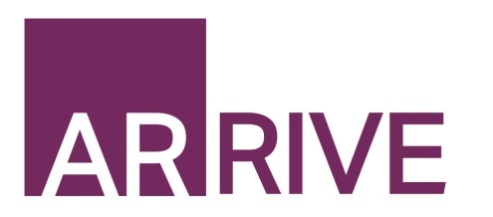


The ARRIVE Guidelines Checklist

Animal Research: Reporting In Vivo Experiments

Carol Kilkenny^1^, William J Browne^2^, Innes C Cuthill^3^, Michael Emerson^4^ and Douglas G Altman^5^

*^1^The National Centre for the Replacement, Refinement and Reduction of Animals in Research, London, UK, ^2^School of Veterinary Science, University of Bristol, Bristol, UK, ^3^School of Biological Sciences, University of Bristol, Bristol, UK, ^4^National Heart and Lung Institute, Imperial College London, UK, ^5^Centre for Statistics in Medicine, University of Oxford, Oxford, UK.*

|  | ITEM | RECOMMENDATION | | Section/ Paragraph |
| --- | --- | --- | --- | --- |
| 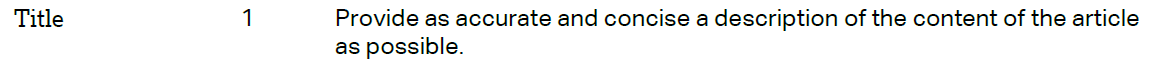 | | | A gain-of-function mutation in the *Plcg2* gene protects mice from *Helicobacter felis*-induced gastric MALT lymphoma by regulatory T- cells | |
| 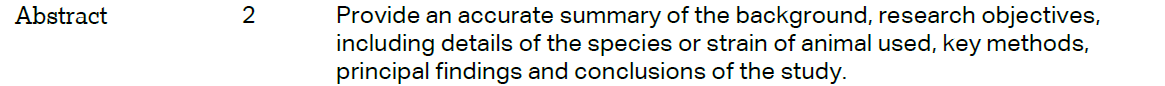 | | | Background & Purpose:  Gastric mucosa-associated lymphoid tissue (MALT) lymphomas develop from a chronic *Helicobacter* infection. *Phospholipase C gamma 2* (PLCG2) is important for B-cell survival and proliferation. We used BALB/c mice with a gain-of-function mutation in the *Plcg2* gene *(Ali5)* to analyze its role in the development of gastric MALT lymphoma*.*  Experimental Approach:  Heterozygous BALB/c *Plcg2^Ali5/+^* and wildtype (WT) mice were orally infected with *H. felis* (2 x 10^8^ bacteria / mouse) on day 1, 3 and 5. Mice were analyzed 6, 12 and 16 months after infection for development of MALT lymphomas. Control groups received no bacteria. To assess proinflammatory cytokines in peripheral blood we used a RT PCR Array (Group 1, followed-up for 6 months). 12 weeks after infection, blood sera were collected to determine antibody titers by ELISA. In uninfected mice, numbers of regulatory T-cells (Tregs) were analyzed in spleen tissue by immunohistochemistry. Mouse spleen mononuclear cells (MNCs) were used to assess CD73 expressing Tregs by flow cytometry. Another group was infected once with 2 x 10^8^ bacteria / mouse every 3rd month for 12 months.  Results:  *Plcg2^Ali5/+^* mice developed less MALT lymphomas than their WT littermates after long-term infection of 16 months. Infected *Plcg2^Ali5/+^* mice showed downregulation of proinflammatory cytokines and decreased *H. felis*-specific IgG1 and IgG2a antibody responses. Additionally, uninfected *Plcg2^Ali5/+^* mice possessed higher numbers of CD73 expressing Tregs.  Conclusion:  These results show a blunted immune response of *Plcg2^Ali5/+^* mice towards *H. felis* infection. Intriguingly, *Plcg2^Ali5/+^* mice possessed higher numbers of CD73 expressing Tregs that might suppress the immune response to *Helicobacter* infection. We suggest that *Plcg2^Ali5/+^* mice may be protected from developing gastric MALT lymphomas as a result of elevated Treg numbers, reduced response to *H. felis* and decrease of proinflammatory cytokines. | |
| INTRODUCTION | | |  | |
| 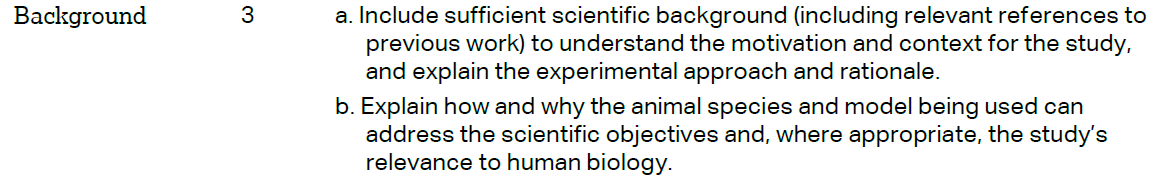 | | | a. Mucosa-associated lymphoid tissue (MALT) lymphomas are extranodal marginal zone B-cell lymphomas. There is a strong association between *Helicobacter pylori* *(H. pylori)* infection and MALT lymphoma, since *H. pylori* is detectable in 92-98% of gastric MALT lymphomas [[1](#_ENREF_1), [2](#_ENREF_2)] and cure of the infection leads to long-lasting remissions [[3-7](#_ENREF_3)].  Several studies showed that different host gene polymorphisms in genes such as *IL-1*, *CTLA4* and *GST T1* are associated with increased inflammatory responses and consecutively with higher incidence of MALT lymphomas [[8-11](#_ENREF_8)]. Previously, our group found that *phospholipase C gamma 2 (PLCG2)* is overexpressed in MALT lymphoma tissue [[12](#_ENREF_12)]. In normal B-cells, B-cell receptor (BCR) antigen-binding results in PLCγ2 phosphorylation by Syk (spleen tyrosine kinase) and Btk (Bruton´s tyrosine kinase). Phosphorylated PLCγ2 is able to cleave phosphatidylinositol 4,5-bisphosphate (PIP_2_) into the second messengers inositol 1,4,5-trisphosphate (IP_3_) and diacylglycerol (DAG) [[13](#_ENREF_13)]. IP_3_ is responsible for calcium release from the endoplasmatic reticulum, while DAG activates PKCβ (protein kinase C) and results in regulation of NF-κB and Ras signaling [[13-16](#_ENREF_13)]. In turn, activation of NF-κB is responsible for cell differentiation, proliferation, survival and development of B-cells [[15](#_ENREF_15), [17](#_ENREF_17)].  b. The mouse strain *Plcg2^Ali5^ (Ali5, abnormal limb 5)* has a genomic *gain-of-function* mutation in the *Plcg2* gene, which results in Plcγ2 hyperactivity due to enhanced membrane adherence after BCR activation [[18](#_ENREF_18)]. This single point-mutation leads to symptoms of systemic inflammatory autoimmune diseases in *Plcg2^Ali5^* mice, which show spontaneous swollen and inflamed paws and autoimmune lupus like disease symptoms, depending on the genetic background. | |
| 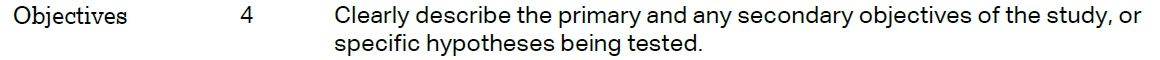 | | | In the present study, we examined the role of a gain-of-function mutation in the *Plcg2* gene in reference to the development of gastric B-cell lymphomas of the MALT-type. We anticipated that due to their autoimmune-prone phenotype, mice with the mutated *Plcg2* gene are more susceptible to development of gastric MALT lymphomas. | |
| METHODS | | |  | |
| 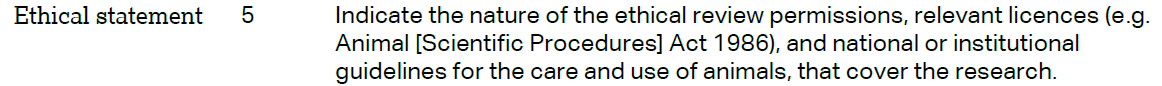 | | | All animal experiments were performed in compliance with the German animal protection law. The study titled “*Helicobacter felis*-induced gastric MALT lymphoma in mice with a gain-of-function mutation in the *PLCg2* gene (MALT lymphoma development)”, were perfomed in approval with institutional guidelines and permissions by the local ethics committee (Regierungspräsidium Gießen) of the state of Hessen, Germany, under the permit numbers V54-19c 20-15(1) MR 20/11 - Nr. 21/2009 and V54-19c 20 15h 01 MR 20/36 Nr. 77/2012. All efforts were made to minimize animal suffering and mice were killed by cervical dislocation. | |
| 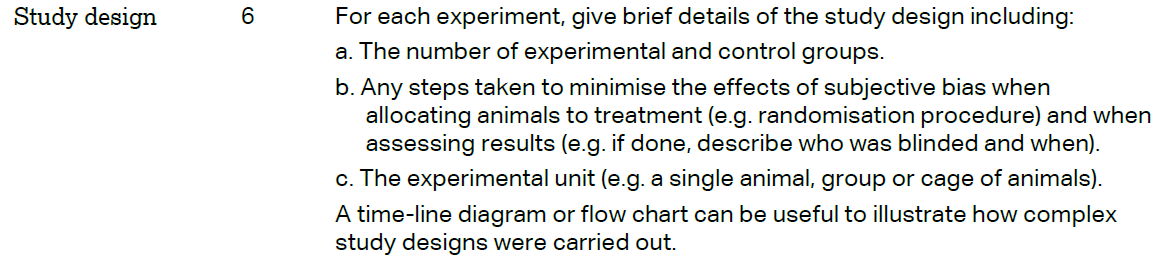 | | | a. MALT lymphoma development:  Table 1 and 2, control group n = 11 per genotype  RT PCR Array:  Group1 (6 months after infection) - uninfected mice: n = 4 per  genotype; infected mice: n = 7 per genotype  B-cell proliferation Assay:  Infected mice: n = 6 per genotype; uninfected mice: n = 3 per  genotype  IgG1 class switching through stimulation of B-cells of non-infected  mice: n = 6 mice per genotype  Immunoglobulin ELISA:  total IgG1, total IgG2a, *H. felis*-specific IgG1 and IgG2a: n = 14  infected tested *Plcg2^Ali5/+^* mice, total IgE: n = 15 infected  *Plcg2^Ali5/+^* mice and n = 15 infected WT mice. Uninfected  control mice: n = 4 per genotype  Histological scoring of Foxp3^+^ Tregs in spleen of uninfected mice.  *Plcg2^Ali5/+^* and WT mice: n = 5 per genotype  Flow cytometry analysis of Tregs and ecto-5`-nucleotidase (CD73)  expressing Tregs of CD4^+^ spleen from uninfected mice: n = 4 per  genotype  b. Immunohistochemical stainings for MALT lymphoma diagnosis  were reviewed in a blinded manner by a reference pathologist.  The total number of Foxp3^+^ Tregs in spleen tissue, was determined  microscopically in all cases by a single investigator to warrant  homogeneity of data collection.  c. ”n” refers to the number of animals. | |
| 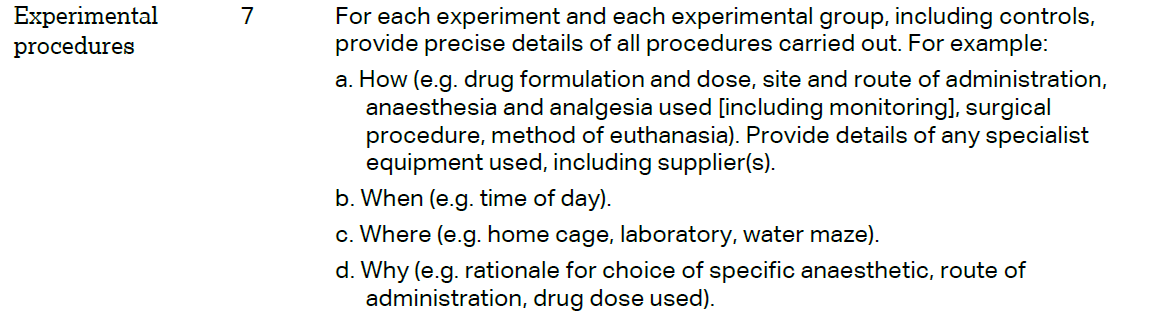 | | | BALB/c *Plcg2^Ali5/+^* and wildtype (WT) mice were orally infected with *H. felis* (2 x 10^8^ bacteria / mouse) by gavage on day 1, 3 and 5. Mice were narcotized with CO_2_ before cervical dislocation 6, 12 and 16 months after infection for development of MALT lymphomas. Control groups received no bacteria. | |
| 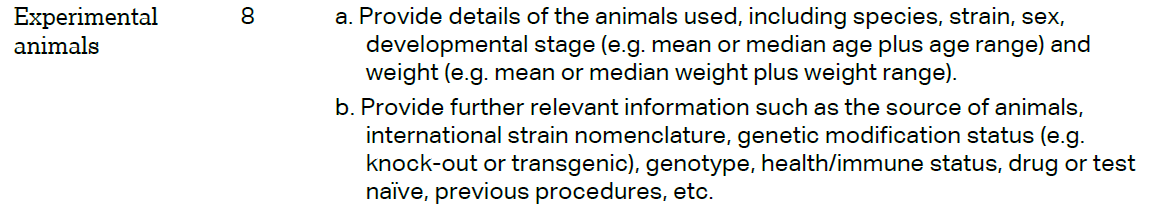 | | | a. Group 1 - 3 (followed-up for 6, 12 and 16 months): female BALB/c WT mice and BALB/c *Plcg2^Ali5^* mice (n = 44 per genotype) were gavaged after they were 8 weeks old.  Group 4 (reinfected group): BALB/c WT mice (n = 3 per gender) and BALB/c *Plcg2^Ali5^* mice (male n = 2, female n = 4) were gavaged after they were 8 weeks old.  b. BALB/c wildtype (WT) mice were purchased from Harlan Winkelmann GmbH. *Plcg2^Ali5/+^* mice with BALB/c background were kindly provided by the Institute of Immunology (Philipps-University Marburg, Germany). | |
|  | | |  | |

The ARRIVE guidelines. Originally published in *PLoS Biology*, June 2010^1^

| 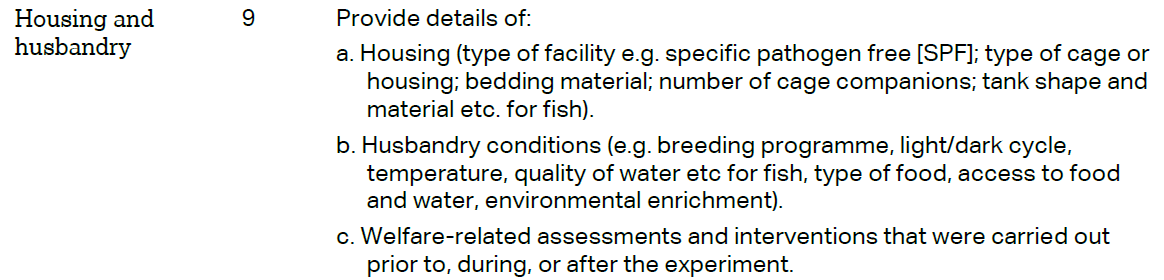 | a. Animals were housed under standardized and SPF conditions. Up  to 6 mice were housed in IVC type II long cages filled with wood  shavings.  b. Environmental conditions were a temperature of 22±1°C,  humidity of 55±5% and 12 hours day-night cycle with lights on at  7:00am. Animals had free access to autoclaved water and pellet  food (Rod 18-R; LASvendi GmbH, Soest, Germany) was  continuously available  c. During housing, animals were monitored 2-3 times a week for health  status (e.g. piloerection, reduction of weight) | |  |
| --- | --- | --- | --- |
| 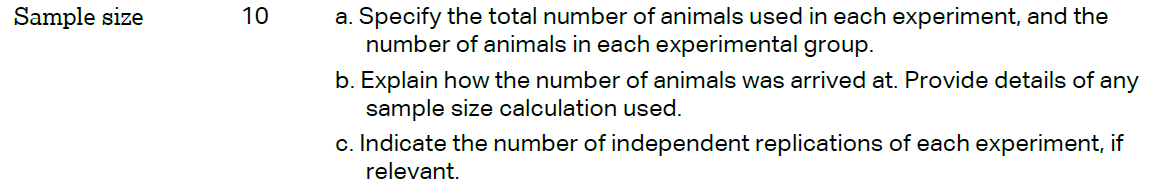 | a. MALT lymphoma development: Table 1, Table 2  b. The number of animals were selected arbitrarily  c. / | |  |
| 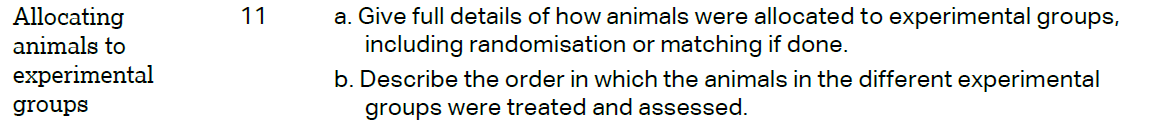 | a. Mice were bred and healthy individuals with almost the same age  were used for infection experiments.  b. For infection experiments, mice with the longest follow-up of 16  months (group 3) was infected first, followed by group 2 (follow-up of  12 months) and group 1 (follow-up of 6 months). | |  |
| 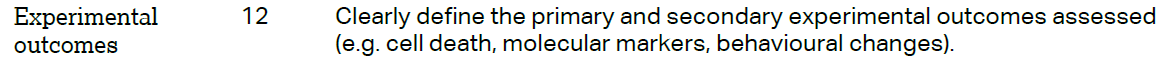 | Mice were cervical dislocated (after CO_2_ narcotization) after a pre-defined infection time. | |  |
| 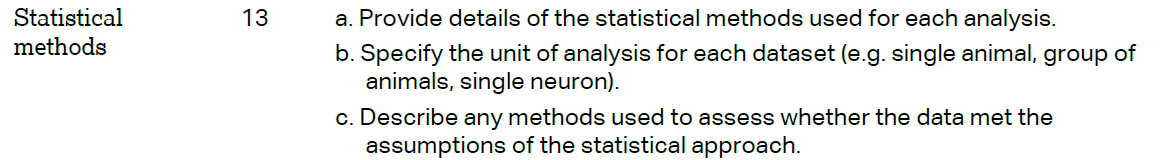 | a. Differences in MALT lymphoma incidence was calculated with  Fisher´s Exact Test. PCR array data were evaluated by the web-  based analysis tool of SABioscience/Qiagen GmbH and calculated  with t-test or two-way analysis of variance (ANOVA) (<http://www.R->  project.org). Statistical analysis of two-tailed t-test (tests for B-cell  function, Foxp3 immunhistochemistry and FACS analysis in spleen  tissue) or Mann-Whitney U-test (ELISA results) was performed with  GraphPad Prism software version 5.01 (GraphPad Software, Inc.).  Statistical significance was assigned at p ≤ 0.05.  b. For each test, the experimental unit was a group of animals  (*Plcg2^Ali5/+^* versus WT)  c. not applicable | |  |
| RESULTS |  | |  |
| 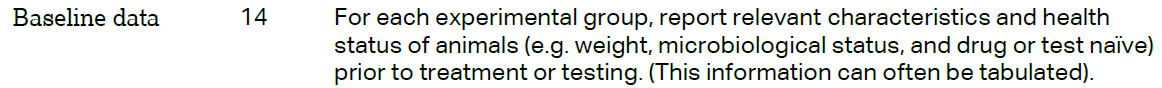 | With the help of a scoring system, all infected animals were monitored 2-3 times a week for health status (e.g. piloerection, reduction of weight). | |  |
| 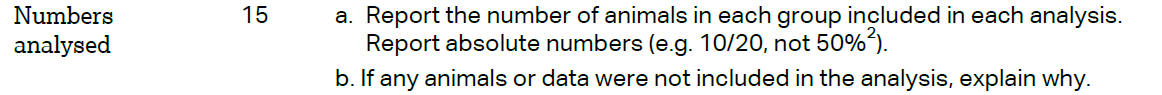 | a. group 1 (follow-up of 6 months):  *Plcg2^Ali5/+^* mice: 10/11  WT Mice: 11/11  group 2 (follow-up of 12 months):  *Plcg2^Ali5/+^* mice: 15/16  WT Mice: 15/16  group 3 (follow-up of 16 months):  *Plcg2^Ali5/+^* mice: 14/17  WT Mice: 15/17  b. Animals which died or have been killed due to the health status  during the study (within 6 months after infection) were not included  in the analysis | |  |
| 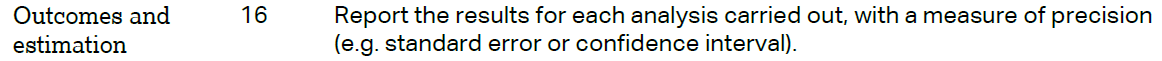 | Paragraphs 1-3 and 6-8, Figures 1-4 and Tables 1-3 | |  |
| 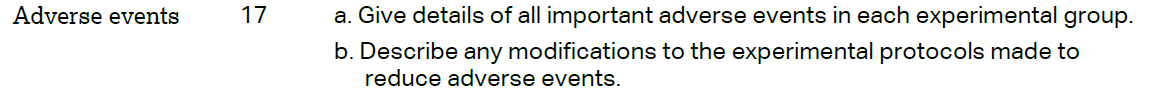 | a. not applicable  b. not applicable | |  |
| DISCUSSION |  | |  |
| 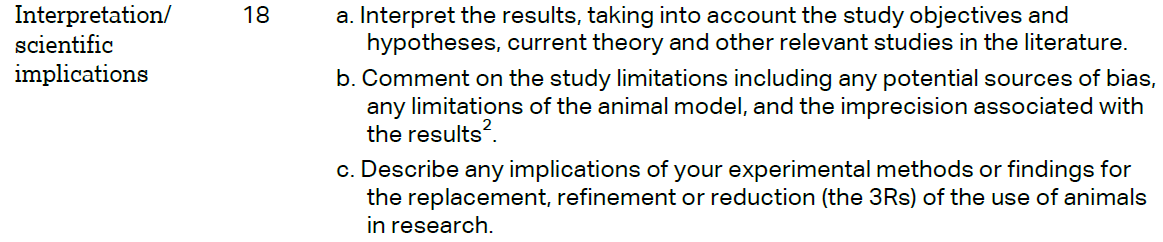 | a. We hypothesized that due to their autoimmune-prone phenotype,  mice with the mutated *Plcg2* gene were more susceptible to  development of gastric MALT lymphomas. However, in contrast to  our hypothesis, we observe less frequent transformation into MALT  lymphomas in BALB/c *Plcg2^Ali5/+^* mice as compared to wild-type  (WT) littermates, and describe that this phenomenon correlates with  impaired immune response, and elevated numbers of suppressive  regulatory T-cells (Tregs). One of the most important factors for  suppression of T-cell-mediated immune responses is the enzyme  ecto-5`-nucleotidase (CD73). CD73 is expressed particularly by  Tregs, but also by tumor cells, myeloid-derived and endothelial cells.  This enzyme is known to generate anti-inflammatory adenosine that  protects tissue damage [[19-23](#_ENREF_19)]. The importance of CD73 expression  was demonstrated in a study by Alam and colleagues [[24](#_ENREF_24)]. They  showed that *H. felis* infected CD73-deficient mice have increased  levels of proinflammatory cytokines and severe gastritis [[24](#_ENREF_24)]. These  data are in line with our findings, that *Plcg2^Ali5/+^* mice had not only  higher numbers of CD4^+^/CD25^+^/Foxp3^+^ Tregs in spleen tissue as  compared to WT mice, but also higher numbers of CD73^+^ Tregs.  Our results support the hypothesis of Kaparakis et al. that Tregs  have a potential role in inhibition of lymphoma development after a  lengthy infection time with *Helicobacter.* Although *Helicobacter spp.*  induce specific immune responses, the immune system of the host  often fails to clear the infection and the bacterium can persist  lifelong. Interaction of bacterial virulence factors and specific  immune answer of the host influence the outcome of the infection.  Several studies showed that different host gene polymorphisms in  genes such as IL-1, CTLA4 and GST T1 are associated with  increased inflammatory responses and consecutively with higher  incidence of MALT lymphomas [[8-11](#_ENREF_8)]. Our mouse model shows for  the first time that a gene polymorphism could also be responsible for  protection from MALT lymphoma development.  b. not applicable  c. Since gastric MALT lymphoma development is strongly dependent  on *Helicobacter* infection, it is not possible to reduce the use of  animals due to our findings. | |  |
| 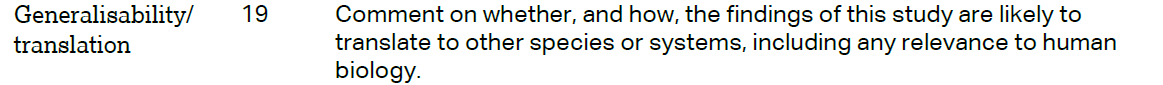 | The *Plcg2* gain-of-function mutation in *Plcg2^Ali5/+^* mice seems to be protective for the development of *H. felis*-induced MALT lymphomas in due to elevated Treg numbers. Based on the facts, that *Helicobacter pylori* (H.p.) colonizes the human gastric mucosa of at least half of the world´s human population [[25](#_ENREF_25), [26](#_ENREF_26)] and different host gene polymorphisms are associated with increased inflammatory responses and higher incidence of MALT lymphomas [[8-11](#_ENREF_8)], it is important to find out how to protect humans against MALT lymphoma development. | |  |
| 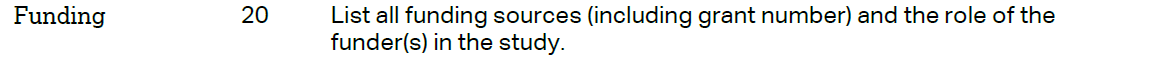 | | This work was supported by Landes-Offensive zur Entwicklung Wissenschaftlich-ökonomischer Exzellenz (LOEWE) grant “Tumor and Inflammation” (project A1, to AN); research grant of the University Hospital Giessen and Marburg (UKGM, 11/2013 MR, to JG and MQH); Deutsche Forschungsgemeinschaft (KFO 210, NE 310/14-2, to AN and SFB TR22, 491.000., to ML); German José Carreras foundation (AH 06-01, to AN); Behring-Röntgen-Stiftung (TP3, 51-0057, to AN and fellowship, 600.000., to ML). | |


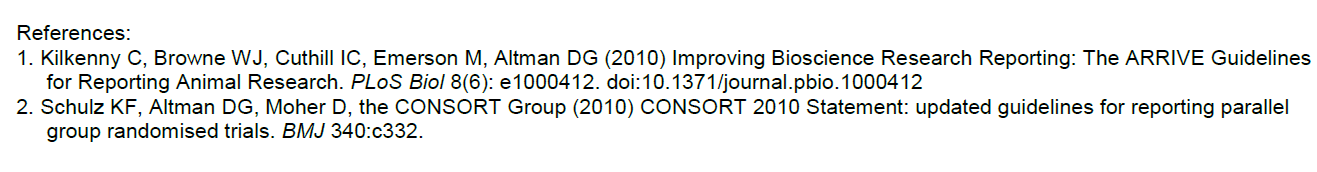

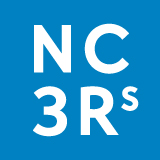


1. Wotherspoon AC, Ortiz-Hidalgo C, Falzon MR, Isaacson PG. Helicobacter pylori-associated gastritis and primary B-cell gastric lymphoma. Lancet. 1991;338(8776):1175-6. Epub 1991/11/09. PubMed PMID: 1682595.

2. Stolte M. Helicobacter pylori gastritis and gastric MALT-lymphoma. Lancet. 1992;339(8795):745-6. Epub 1992/03/21. PubMed PMID: 1347613.

3. Wündisch T, Dieckhoff P, Greene B, Thiede C, Wilhelm C, Stolte M, et al. Second cancers and residual disease in patients treated for gastric mucosa-associated lymphoid tissue lymphoma by Helicobacter pylori eradication and followed for 10 years. Gastroenterology. 2012;143(4):936-42. Epub 2012/07/04. doi: 10.1053/j.gastro.2012.06.035. PubMed PMID: 22750463.

4. Bayerdörffer E, Neubauer A, Rudolph B, Thiede C, Lehn N, Eidt S, et al. Regression of primary gastric lymphoma of mucosa-associated lymphoid tissue type after cure of Helicobacter pylori infection. MALT Lymphoma Study Group. Lancet. 1995;345(8965):1591-4. Epub 1995/06/24. PubMed PMID: 7783535.

5. Wündisch T, Thiede C, Morgner A, Dempfle A, Günther A, Liu H, et al. Long-term follow-up of gastric MALT lymphoma after Helicobacter pylori eradication. Journal of clinical oncology : official journal of the American Society of Clinical Oncology. 2005;23(31):8018-24. Epub 2005/10/06. doi: 10.1200/JCO.2005.02.3903. PubMed PMID: 16204012.

6. Wündisch T, Mosch C, Neubauer A, Stolte M. Helicobacter pylori eradication in gastric mucosa-associated lymphoid tissue lymphoma: Results of a 196-patient series. Leukemia & lymphoma. 2006;47(10):2110-4. Epub 2006/10/31. doi: 10.1080/10428190600783536. PubMed PMID: 17071484.

7. Neubauer A, Thiede C, Morgner A, Alpen B, Ritter M, Neubauer B, et al. Cure of Helicobacter pylori infection and duration of remission of low-grade gastric mucosa-associated lymphoid tissue lymphoma. Journal of the National Cancer Institute. 1997;89(18):1350-5. Epub 1997/10/06. PubMed PMID: 9308704.

8. Hellmig S, Vollenberg S, Goebeler-Kolve ME, Fischbach W, Hampe J, Folsch UR, et al. IL-1 gene cluster polymorphisms and development of primary gastric B-cell lymphoma in Helicobacter pylori infection. Blood. 2004;104(9):2994-5. Epub 2004/10/23. doi: 10.1182/blood-2004-05-1792. PubMed PMID: 15498865.

9. Rollinson S, Levene AP, Mensah FK, Roddam PL, Allan JM, Diss TC, et al. Gastric marginal zone lymphoma is associated with polymorphisms in genes involved in inflammatory response and antioxidative capacity. Blood. 2003;102(3):1007-11. Epub 2003/04/05. doi: 10.1182/blood-2002-12-3803. PubMed PMID: 12676777.

10. Chiu BC, Kolar C, Gapstur SM, Lawson T, Anderson JR, Weisenburger DD. Association of NAT and GST polymorphisms with non-Hodgkin's lymphoma: a population-based case-control study. British journal of haematology. 2005;128(5):610-5. Epub 2005/02/24. doi: 10.1111/j.1365-2141.2004.05358.x. PubMed PMID: 15725081.

11. Cheng TY, Lin JT, Chen LT, Shun CT, Wang HP, Lin MT, et al. Association of T-cell regulatory gene polymorphisms with susceptibility to gastric mucosa-associated lymphoid tissue lymphoma. Journal of clinical oncology : official journal of the American Society of Clinical Oncology. 2006;24(21):3483-9. Epub 2006/07/20. doi: 10.1200/JCO.2005.05.5434. PubMed PMID: 16849765.

12. Huynh MQ, Wacker HH, Wundisch T, Sohlbach K, Kim TD, Krause M, et al. Expression profiling reveals specific gene expression signatures in gastric MALT lymphomas. Leukemia & lymphoma. 2008;49(5):974-83. Epub 2008/05/09. doi: 10.1080/10428190802007734. PubMed PMID: 18464117.

13. Bunney TD, Katan M. PLC regulation: emerging pictures for molecular mechanisms. Trends in biochemical sciences. 2011;36(2):88-96. Epub 2010/09/28. doi: 10.1016/j.tibs.2010.08.003. PubMed PMID: 20870410.

14. Teixeira C, Stang SL, Zheng Y, Beswick NS, Stone JC. Integration of DAG signaling systems mediated by PKC-dependent phosphorylation of RasGRP3. Blood. 2003;102(4):1414-20. Epub 2003/05/06. doi: 10.1182/blood-2002-11-3621. PubMed PMID: 12730099.

15. Bell SE, Vigorito E, McAdam S, Reynolds HM, Caraux A, Colucci F, et al. PLCgamma2 regulates Bcl-2 levels and is required for survival rather than differentiation of marginal zone and follicular B cells. European journal of immunology. 2004;34(8):2237-47. Epub 2004/07/20. doi: 10.1002/eji.200425054. PubMed PMID: 15259021.

16. Petro JB, Khan WN. Phospholipase C-gamma 2 couples Bruton's tyrosine kinase to the NF-kappaB signaling pathway in B lymphocytes. The Journal of biological chemistry. 2001;276(3):1715-9. Epub 2000/10/24. doi: 10.1074/jbc.M009137200. PubMed PMID: 11042193.

17. Nielsen CH, Leslie RGQ. Regulation of B-Cell Activation by Complement Receptors and Fc Receptors. Transfusion Medicine and Hemotherapy. 2005;32(6):339-47. doi: 10.1159/000089121.

18. Yu P, Constien R, Dear N, Katan M, Hanke P, Bunney TD, et al. Autoimmunity and inflammation due to a gain-of-function mutation in phospholipase C gamma 2 that specifically increases external Ca2+ entry. Immunity. 2005;22(4):451-65. Epub 2005/04/23. doi: 10.1016/j.immuni.2005.01.018. PubMed PMID: 15845450.

19. Borsellino G, Kleinewietfeld M, Di Mitri D, Sternjak A, Diamantini A, Giometto R, et al. Expression of ectonucleotidase CD39 by Foxp3+ Treg cells: hydrolysis of extracellular ATP and immune suppression. Blood. 2007;110(4):1225-32. Epub 2007/04/24. doi: 10.1182/blood-2006-12-064527. PubMed PMID: 17449799.

20. Kumar V, Sharma A. Adenosine: an endogenous modulator of innate immune system with therapeutic potential. European journal of pharmacology. 2009;616(1-3):7-15. Epub 2009/05/26. doi: 10.1016/j.ejphar.2009.05.005. PubMed PMID: 19464286.

21. Stagg J, Smyth MJ. Extracellular adenosine triphosphate and adenosine in cancer. Oncogene. 2010;29(39):5346-58. Epub 2010/07/28. doi: 10.1038/onc.2010.292. PubMed PMID: 20661219.

22. Deaglio S, Dwyer KM, Gao W, Friedman D, Usheva A, Erat A, et al. Adenosine generation catalyzed by CD39 and CD73 expressed on regulatory T cells mediates immune suppression. The Journal of experimental medicine. 2007;204(6):1257-65. Epub 2007/05/16. doi: 10.1084/jem.20062512. PubMed PMID: 17502665; PubMed Central PMCID: PMC2118603.

23. Beavis PA, Stagg J, Darcy PK, Smyth MJ. CD73: a potent suppressor of antitumor immune responses. Trends in immunology. 2012;33(5):231-7. Epub 2012/04/11. doi: 10.1016/j.it.2012.02.009. PubMed PMID: 22487321.

24. Alam MS, Kurtz CC, Rowlett RM, Reuter BK, Wiznerowicz E, Das S, et al. CD73 is expressed by human regulatory T helper cells and suppresses proinflammatory cytokine production and Helicobacter felis-induced gastritis in mice. The Journal of infectious diseases. 2009;199(4):494-504. Epub 2009/03/14. doi: 10.1086/596205. PubMed PMID: 19281303; PubMed Central PMCID: PMC3047419.

25. Suerbaum S, Michetti P. Helicobacter pylori infection. The New England journal of medicine. 2002;347(15):1175-86. Epub 2002/10/11. doi: 10.1056/NEJMra020542. PubMed PMID: 12374879.

26. Frenck RW, Jr., Clemens J. Helicobacter in the developing world. Microbes and infection / Institut Pasteur. 2003;5(8):705-13. Epub 2003/06/20. PubMed PMID: 12814771.
