# Supplementary figures and images for: A Gain-Of-Function Mutation in the Plcg2 Gene Protects Mice from Helicobacter felis-Induced Gastric MALT Lymphoma
Source: PLoS One. 2016 Mar 11;11(3):e0150411. doi: 10.1371/journal.pone.0150411 (PMC4788355; doi:10.1371/journal.pone.0150411)

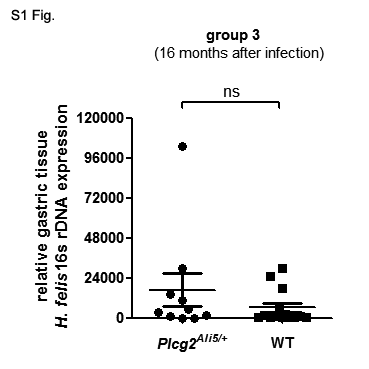

Supplement: S1 Fig — The bacterial load in gastric tissue of Plcg2Ali5/+ (n = 10) and WT mice (n = 12) was measured by quantitative Real-Time PCR, 16 months after H. felis infection. Data represent mean ± SEM and were calculated using Student´s t-test. ns = not significant. (TIF) [file pone.0150411.s002.tif]

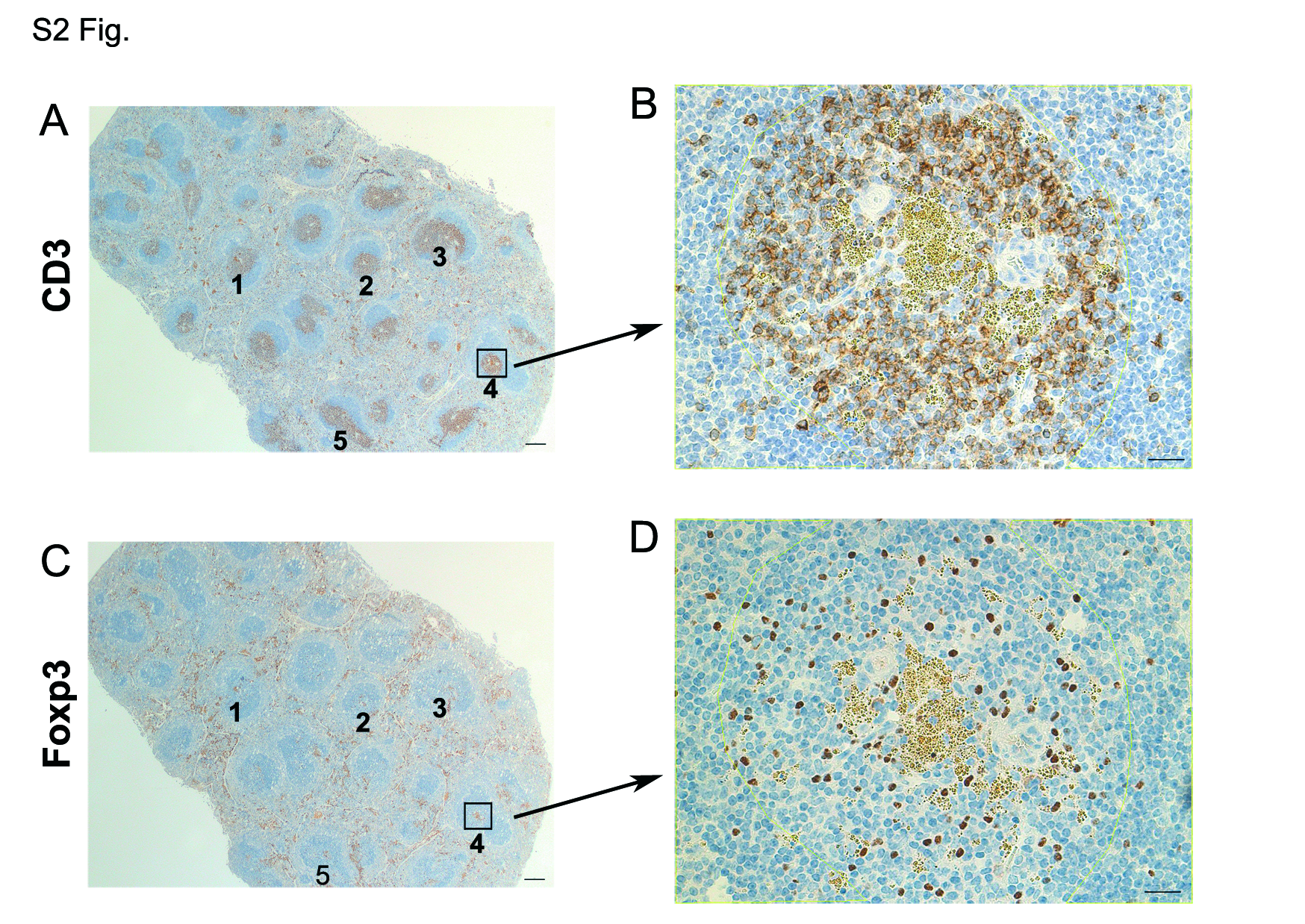

Supplement: S2 Fig — A representative (A-B) CD3 and (C-D) Foxp3 immunohistochemical staining of a spleen of an uninfected mouse. The same five T-cell areas (in CD3 and Foxp3 staining) of the white pulp were determined microscopically and total Foxp3+ Tregs were determined with a magnification of x40. By using ImageJ v1.47 software total CD3 area was calculated (thin yellow line) and Foxp3+ Tregs were determined in relation to 1000 μm2 (CD3+ T-cell area). (A and C) Scale bars show 200 μm with an original magnification of x2,5 or (B and D) 20 μm with an magnification of x40. (TIF) [file pone.0150411.s003.tif]
