# Supplementary material for: A Gain-Of-Function Mutation in the Plcg2 Gene Protects Mice from Helicobacter felis-Induced Gastric MALT Lymphoma
Source: PLoS One. 2016 Mar 11;11(3):e0150411. doi: 10.1371/journal.pone.0150411 (PMC4788355; doi:10.1371/journal.pone.0150411)
Supplement: S1 Materials and Methods — (DOCX) [file pone.0150411.s004.docx]

**SI Materials and Methods**

## Quantitative Real-Time PCR

Quantitative PCR was performed as previously described [1]. Mouse PCR primers were: GAPDH forward 5`-CATGGCCTTCCGTGTTCCTA-3´ and reverse 5`-CCTGCTTCACCACCTTCTTGA-3`; Tnfrsf13c forward 5`- CCGCACTCCTGGGACTGATACTG-3` and reverse 5`-TCCTGAGCTGTTGACGCCACC-3`; S100A8 forward 5`-GGAGTTCCTTGCGATGGTGATAAAAG-3` and reverse 5`-TACTCCTTGTGGCTGTCTTTGTGAG-3`. *H. felis* specific 16S rDNA primers were: CAR557.for 5`-TGCGTAGGCGGGGTTGTAAG-3` and CAR636.rev 5`-CAGAGTTGTAGTTTGAAATGC-3`. Primers were obtained from Eurofins MWG Operon (Ebersberg, Germany).

## Assessment of *H. felis* colonization in gastric tissue

To assess the bacterial load in gastric tissue we performed the quantitative PCR method. We used *H. felis* specific 16S rDNA primers, which were previously described [2]. For quantification we used inactivated bacteria (56°C for 10 min) as standard curve (10^2^ to 10^7^ bacteria/ml) and 900 ng genomic DNA of mouse gastric tissue. DNA was isolated using the Allprep RNA/DNA Kit (Qiagen, Hilden, Germany) according to the manufacturer’s instructions. Mean values of triplicates were used for calculation of *H. felis* colonization in mice group 3 (16 months after infection).

1. Huynh MQ, Wacker HH, Wundisch T, Sohlbach K, Kim TD, Krause M, et al. Expression profiling reveals specific gene expression signatures in gastric MALT lymphomas. Leukemia & lymphoma. 2008;49(5):974-83. Epub 2008/05/09. doi: 10.1080/10428190802007734. PubMed PMID: 18464117

2. De Groote D, Haesebrouck F, van Doorn LJ, Vandamme P, Ducatelle R. Evaluation of a group-specific 16S ribosomal DNA-based PCR for detection of Helicobacter bizzozeronii, Helicobacter felis, and Helicobacter salomonis in fresh and paraffin-embedded gastric biopsy specimens. Journal of clinical microbiology. 2001;39(3):1197-9. Epub 2001/03/07. doi: 10.1128/JCM.39.3.1197-1199.2001. PubMed PMID: 11230459; PubMed Central PMCID: PMC87905.
