# Supplementary material for: A Gain-Of-Function Mutation in the Plcg2 Gene Protects Mice from Helicobacter felis-Induced Gastric MALT Lymphoma
Source: PLoS One. 2016 Mar 11;11(3):e0150411. doi: 10.1371/journal.pone.0150411 (PMC4788355; doi:10.1371/journal.pone.0150411)
Supplement: S1 Table — (DOCX) [file pone.0150411.s005.docx]

**Table S1: Scoring System**

| **Parameter** | | **Points** |
| --- | --- | --- |
| **Appearance** | Normal | **0** |
|  | Lack of grooming | **1** |
|  | Eye and nasal discharge | **2** |
|  | Piloerection | **3** |
| **Water and feed intake** | Normal | **0** |
|  | Weight loss <5% within 10 days | **1** |
|  | Weight loss <10-15% within 5 days | **2** |
|  | No water and feed intake | **3** |
| **Clinical signs** | Normal breathing | **0** |
|  | Slight changes | **1** |
|  | Increased respiratory rate with slight abdominal breathing | **2** |
|  | Abdominal breathing | **3** |
| **Unaffected behaviour** | Normal | **0** |
|  | Slight conspicuousness | **1** |
|  | Slight disabilities | **2** |
|  | Isolation and very quiet | **3** |
| **Triggered behaviour** | Normal | **0** |
|  | Mild depression or exaggerated response | **1** |
|  | Mild changes in behaviour | **2** |
|  | Weak reaction | **3** |

**Evaluation (Points):**  **0 - 1** Normal

**2 - 3** Observation once per day

**≥ 4**  Observation (at least twice per day)

**6** Veterinary expertise

(Take in consideration: Temgesic 0,05-0,1 mg/kg)

**≥ 7**  Decision for euthanasia

**≥ 9** Euthanasia (if weight loss is more than 20% or incidence of 2 parameters with award of 3 points)
