# Supplementary material for: A Gain-Of-Function Mutation in the Plcg2 Gene Protects Mice from Helicobacter felis-Induced Gastric MALT Lymphoma
Source: PLoS One. 2016 Mar 11;11(3):e0150411. doi: 10.1371/journal.pone.0150411 (PMC4788355; doi:10.1371/journal.pone.0150411)
Supplement: S2 Table — (DOCX) [file pone.0150411.s006.docx]

**Table S2:** **Histopathological results of uninfected Balb/c *Plcg2^Ali5/+^* and WT mice.**

| **control mice** | **Genotype** | **Number of mice** | | **Normal findings** |
| --- | --- | --- | --- | --- |
|  |  |  | |  |
|  |  | **Enrolled^a^** | **Died^b^** |  |
| **uninfected** | *Plcg2 ^Ali5/+^* | 11 | 0 | 100%  (11/11) |
|  | WT | 11 | 1 | 100%  (10/10) |

^a^ Number of mice enrolled at the beginning of the study.

^b^ Number of mice that died/killed during the study (within 6 months after infection).
