# Supplementary material for: A Gain-Of-Function Mutation in the Plcg2 Gene Protects Mice from Helicobacter felis-Induced Gastric MALT Lymphoma
Source: PLoS One. 2016 Mar 11;11(3):e0150411. doi: 10.1371/journal.pone.0150411 (PMC4788355; doi:10.1371/journal.pone.0150411)
Supplement: S3 Table — (DOCX) [file pone.0150411.s007.docx]

**S3 Table:** Proinflammatory cytokine profile of *Plcg2^Ali5/+^* and WT mice (either uninfected or 6 months after *H. felis* infection) grouped in genotype and infection status.

| Status | Genes | Direction | *P*-value |
| --- | --- | --- | --- |
| Genotype^a^ | Ccl3 | Down | 0.0047 |
|  | Cd40 | Down | 0.0378 |
|  | Cxcr1 | Up | 0.0175 |
|  | S100a8 | Up | 0.0030 |
|  | Tlr2 | Up | 0.0100 |
|  | Tnfrsf13c | Down | 0.0002 |
| Infection^b^ | Cxcl5 | Down | 0.0119 |
|  | Tnfrsf13c | Down | 0.0131 |

Significant alterations in expression of proinflammatory genes in whole peripheral blood were analyzed by two-way ANOVA (*p ≤ .05* and fold regulation of at least ± 2).

^a^ Genotype status, independent on *H. felis* infection (*Plcg2^Ali5/+^* versus WT mice)

^b^ Infection status, independent on genotype status (infected versus uninfected mice)
