# Supplementary material for: A Gain-Of-Function Mutation in the Plcg2 Gene Protects Mice from Helicobacter felis-Induced Gastric MALT Lymphoma
Source: PLoS One. 2016 Mar 11;11(3):e0150411. doi: 10.1371/journal.pone.0150411 (PMC4788355; doi:10.1371/journal.pone.0150411)
Supplement: S4 Table — (DOCX) [file pone.0150411.s008.docx]

**Table S4:** PCR array validation by quantitative PCR.

| **Gene** | **Status** | **Experiment** | **Fold change** | **p-value** |
| --- | --- | --- | --- | --- |
| **Tnfrsf13c** | **Infection** | **PCR Array** | **-1,8483** | 0,013 (two-way ANOVA) |
|  |  |  |  | 0,004 (test) |
|  |  | **Quantitative PCR** | **-2,349** | 0,036 (t-test) |
|  | **Genotype** | **PCR Array** | **-2,786** | 0,00015 (two-way ANOVA) |
|  |  |  |  | 0,014 (t-test) |
|  |  | **Quantitative PCR** | **-2,266** | 0,046 (t-test) |
| **S100a8** | **Genotype** | **PCR Array** | **2,614** | 0,003 (two-way ANOVA) |
|  |  |  |  | 0,014 (t-test) |
|  |  | **Quantitative PCR** | **3,418** | 0,018 (t-test) |

Quantitative real-time PCR was performed to validate the Array data. Comparison of qPCR and array data for **(A)** genotype

and **(B)** infection status.
